# Apparent Lack of *BRAF*^*V600E*^ Derived HLA Class I Presented Neoantigens Hampers Neoplastic Cell Targeting by CD8^+^ T Cells in Langerhans Cell Histiocytosis

**DOI:** 10.3389/fimmu.2019.03045

**Published:** 2020-01-10

**Authors:** Paul G. Kemps, Timo C. Zondag, Eline C. Steenwijk, Quirine Andriessen, Jelske Borst, Sandra Vloemans, Dave L. Roelen, Lenard M. Voortman, Robert M. Verdijk, Carel J. M. van Noesel, Arjen H. G. Cleven, Cynthia Hawkins, Veronica Lang, Arnoud H. de Ru, George M. C. Janssen, Geert W. Haasnoot, Kees L. M. C. Franken, Ronald van Eijk, Nienke Solleveld-Westerink, Tom van Wezel, R. Maarten Egeler, Auke Beishuizen, Jan A. M. van Laar, Oussama Abla, Cor van den Bos, Peter A. van Veelen, Astrid G. S. van Halteren

**Affiliations:** ^1^Immunology Laboratory Willem-Alexander Children's Hospital, Leiden University Medical Center, Leiden, Netherlands; ^2^Department of Immunology, Erasmus University Medical Center, Rotterdam, Netherlands; ^3^Department of Immunohematology and Blood Transfusion, Leiden University Medical Center, Leiden, Netherlands; ^4^Department of Cell and Chemical Biology, Leiden University Medical Center, Leiden, Netherlands; ^5^Department of Pathology, Erasmus University Medical Center, Rotterdam, Netherlands; ^6^Department of Pathology, Amsterdam University Medical Centers, Amsterdam, Netherlands; ^7^Department of Pathology, Leiden University Medical Center, Leiden, Netherlands; ^8^Hospital for Sick Children, University of Toronto, Toronto, ON, Canada; ^9^Center for Proteomics and Metabolomics, Leiden University Medical Center, Leiden, Netherlands; ^10^Department of Pediatric Oncology, Sophia Children's Hospital, Erasmus University Medical Center, Rotterdam, Netherlands; ^11^Princess Máxima Center for Pediatric Oncology, Utrecht, Netherlands; ^12^Department of Pediatric Oncology, Emma Children's Hospital, Amsterdam University Medical Centers, Amsterdam, Netherlands

**Keywords:** Langerhans Cell Histiocytosis, BRAF, neoantigen, neopeptide, Human Leukocyte Antigen, T cell

## Abstract

Langerhans Cell Histiocytosis (LCH) is a neoplastic disorder of hematopoietic origin characterized by inflammatory lesions containing clonal histiocytes (LCH-cells) intermixed with various immune cells, including T cells. In 50–60% of LCH-patients, the somatic *BRAF*^*V600E*^ driver mutation, which is common in many cancers, is detected in these LCH-cells in an otherwise quiet genomic landscape. Non-synonymous mutations like *BRAF*^*V600E*^ can be a source of neoantigens capable of eliciting effective antitumor CD8^+^ T cell responses. This requires neopeptides to be stably presented by Human Leukocyte Antigen (HLA) class I molecules and sufficient numbers of CD8^+^ T cells at tumor sites. Here, we demonstrate substantial heterogeneity in CD8^+^ T cell density in *n* = 101 LCH-lesions, with *BRAF*^*V600E*^ mutated lesions displaying significantly lower CD8^+^ T cell:CD1a^+^ LCH-cell ratios (*p* = 0.01) than *BRAF* wildtype lesions. Because LCH-lesional CD8^+^ T cell density had no significant impact on event-free survival, we investigated whether the intracellularly expressed *BRAF*^*V600E*^ protein is degraded into neopeptides that are naturally processed and presented by cell surface HLA class I molecules. Epitope prediction tools revealed a single HLA class I binding *BRAF*^*V600E*^ derived neopeptide (KIGDFGLAT**E**K), which indeed displayed strong to intermediate binding capacity to HLA-A^*^03:01 and HLA-A^*^11:01 in an *in vitro* peptide-HLA binding assay. Mass spectrometry-based targeted peptidomics was used to investigate the presence of this neopeptide in HLA class I presented peptides isolated from several *BRAF*^*V600E*^ expressing cell lines with various HLA genotypes. While the HLA-A^*^02:01 binding *BRAF* wildtype peptide KIGDFGLATV was traced in peptides isolated from all five cell lines expressing this HLA subtype, KIGDFGLAT**E**K was not detected in the HLA class I peptidomes of two distinct *BRAF*^*V600E*^ transduced cell lines with confirmed expression of HLA-A^*^03:01 or HLA-A^*^11:01. These data indicate that the *in silico* predicted HLA class I binding and proteasome-generated neopeptides derived from the *BRAF*^*V600E*^ protein are not presented by HLA class I molecules. Given that the *BRAF*^*V600E*^ mutation is highly prevalent in chemotherapy refractory LCH-patients who may qualify for immunotherapy, this study therefore questions the efficacy of immune checkpoint inhibitor therapy in LCH.

## Introduction

Langerhans Cell Histiocytosis (LCH) is a rare neoplastic disorder of hematopoietic origin that primarily affects children, but also involves adults (1). Its clinical manifestation varies from a single bone lesion or benign skin rash to a widely disseminated and life-threatening condition, similar to acute myeloid leukemia (2). The histopathological hallmark of LCH are phenotypically aberrant CD1a^+^ CD207^+^ histiocytes (LCH-cells), although not all pathological CD1a^+^ histiocytes co-express CD207 (3). Typically, these LCH-cells are accompanied by a diverse inflammatory infiltrate, often including T cells (2). These T cells have been shown to frequently make intimate contact with LCH-cells (4, 5). While patients with high CD8^+^ T cell density in the tumor infiltrate have a more favorable prognosis across many other neoplastic diseases (6), little is still known about the presence and clinical impact of CD8^+^ T cells in LCH-lesions (7–9).

Naive (CD8^+^) T cells require antigen binding by their T cell receptor and co-stimulatory signals for (proper) activation. Previous studies have already demonstrated that LCH-cells express the co-stimulatory receptors CD40 (10–12), CD80 (3, 11–13), ICOS ligand (ICOSL) (14) and, although variably, CD86 (3, 11, 12) *in situ*. Moreover, transcriptome analyses revealed that LCH-cells express similar levels of CD40, CD80, and CD86 messenger RNA when compared to normal epidermal CD207^+^ Langerhans cells (15, 16), and that they confer high expression of genes relevant for antigen presentation (including *CD1E*) and genes encoding members of the HLA (class II) complex (17). Thus, LCH-cells do not appear to have an intrinsic defect in their capacity to elicit a T cell immune response (12). This may explain why a proportion of LCH-lesional T cells have been shown to express cell surface markers indicative of recent activation (2), including CD40L (10), ICOS (14), CXCR3 (7), CD25 (5, 14), PD-1 (18, 19), RANKL (20), and CD45RO (7). In addition, marked monoclonal expansion of LCH-lesion infiltrating CD3^+^ T cells has been observed (19), suggesting that T cell receptor activation occurred *in situ*. The antigen-specificity of activated LCH-lesional T cells is, however, unknown (2).

In 2010, universal activation of the mitogen-activated protein kinase (MAPK) signaling pathway in LCH-cells was demonstrated (21, 22). Since then, recurrent somatic mutations in genes of the MAPK signaling pathway have been identified in ~85% of LCH-patients (23, 24). Oncogenic driver mutations are essential for tumorigenesis and tend to be clonally conserved. This makes neoantigens derived from proteins encoded by oncogenes highly attractive targets for immunotherapy. In addition, the natural T cell pool should contain T cells expressing high affinity T cell receptors for these neoantigens (25), which may exert potent antitumor function (26–29). This requires, however, neoantigens to be stably presented by Human Leukocyte Antigen (HLA) class I molecules and sufficient numbers of CD8^+^ T cells at tumor sites. Over the past years, several HLA class I presented “public” neoantigens resulting from recurrent hotspot mutations in driver oncogenes have been discovered (30–38). Approximately 50–60% of LCH-patients carry the somatic *BRAF*^*V600E*^ driver mutation (1, 21). CD8^+^ T cells specific for *BRAF*^*V600E*^ derived neopeptides have already been reported *in vitro* and in murine models (39–42). Thus, activation of LCH-lesional *BRAF*^*V600E*^ neoantigen-specific CD8^+^ T cells could hypothetically lead to the eradication of *BRAF*^*V600E*^ expressing LCH-cells. Moreover, the concurrent formation of long-lasting bone-marrow homing memory CD8^+^ T cells could control new outgrowth of residual *BRAF*^*V600E*^ mutated histiocyte precursor cells (43). Immunotherapy specifically aimed at enhancing the number and effector function of these *BRAF*^*V600E*^-specific CD8^+^ T cells could offer great promise in the treatment of high-risk LCH-patients, given that these patients often bear the *BRAF*^*V600E*^ mutation and fail first-line chemotherapy (44). Importantly, the *BRAF* gene is mutated in ~7% of human cancers, with the *BRAF*^*V600E*^ mutation accounting for >90% of all genetic variations (45, 46). Hence, the identification of HLA class I presented “public” neoantigens derived from the *BRAF*^*V600E*^ protein would offer great therapeutic opportunity for many patients with other *BRAF*^*V600E*^ mutated neoplasms as well (47).

The aim of this study was therefore to (i) assess the presence and clinical impact of lesional CD8^+^ T cells in (HLA and BRAF^*V600E*^) genotyped LCH-patients, and (ii) to investigate whether *BRAF*^*V600E*^ derived neopeptides are presented by HLA class I molecules and could be recognized by such CD8^+^ T cells.

## Materials and Methods

### Patients and Samples

Patient accrual started after approval of the study protocol (CCMO NL33428.058.10) by each local Institutional Review Board. Only patients of whom formalin-fixed-paraffin-embedded (FFPE) first disease onset (FDO) LCH tissue biopsies were available were asked to participate in the study. Informed consent was provided by *n* = 135 patients and/or their parents/legal guardians. LCH diagnosis was confirmed by a combination of clinical findings and the presence of phenotypically aberrant CD1a^+^ histiocytes in the tissue biopsy. The tissue samples were handled according to the code of conduct for proper secondary use of human tissue of the Federation of Dutch Medical Scientific Societies (FEDERA). Clinical information was collected by each participating center separately using a standardized Case Report Form (CRF) and anonymized data were provided to the researchers of the LUMC. Events were defined as LCH disease progression or reactivation. Progression was defined as (i) progression of existing lesions requiring start or intensification of systemic chemotherapy and/or radiotherapy, or (ii) the development of new lesions when Non-Active Disease (NAD) state had not yet been attained. LCH reactivation was defined as the development of new lesions after NAD had been attained for LCH FDO.

### Flow Cytometric Analysis of LCH Tissue Biopsies

Fresh LCH tissue was dissociated using a gentle MACS tissue dissociator (Miltenyi Biotec) and single cells were cryopreserved in DMSO and albumin containing Roswell Park Memorial Institute (RPMI) culture medium. Before flow cytometric analysis, cells were thawed in RPMI + 20% fetal calf serum (FCS) + Penicillin-Streptomycin (P/S) containing 1,600 IU/ml DNAase (Sigma-Aldrich). After washing, the cells were stained with a mixture of different antibodies: CD45 (2D1, 1:50, BD Biosciences), CD1a (HI149, 1:50, BD Biosciences), CD207 (DCGM4, 1:25, Beckman Coulter), CD14 (MØP9, 1:20, BD Biosciences), CD3 (UCHT1, 1:200, BD Biosciences), CD8 (SK1, 1:100, BD Biosciences), HLA-DR (G46-6, 1:200, BD Biosciences), and panHLA class I (G46-2.6, 1:40, BD Biosciences). The cells were then re-washed and immediately analyzed on a FACS ARIA3 or FACS Fusion cell sorter (BD Biosciences).

### HLA Genotyping and Analysis

High-resolution HLA genotyping was performed by DKMS Life Sciences Lab on DNA extracted from buccal swabs obtained from *n* = 104 LCH-patients using an ampliqon sequencing-based approach, as previously described (48, 49). For *n* = 14 additional patients, low-resolution HLA genotype data were acquired using a sequence specific oligoprimer-based approach (50). Hardy-Weinberg Equilibrium testing and HLA association analyses were performed using the HLA genotype data of Dutch LCH-patients. To evaluate statistical significance, two-sided Fisher's exact tests were carried out. The *p*-values were corrected for multiple comparisons conform the Šidák method (51). Odds ratios and corresponding 95% confidence intervals were calculated according to the method of Woolf with the Haldane correction (52, 53). Since a large control group could lead to significant differences that are clinically irrelevant, *p*-values were standardized to a smaller control sample size following the method of Good (54). The smaller control sample size was obtained using the following calculation: the total number of LCH-patients plus 3 times the number of patients as maximum allowed size for the control group.

### Immunohistochemical Staining of LCH Tissue Sections

FFPE tissue sections (4–10 μm) were deposited on Superfrost™ (Thermo Fisher Scientific) glass slides, dried overnight at 37°C and stored at 4°C. Prior to immunohistochemical (IHC) staining, selected 4 μm slides were preheated at 66°C for 1 h and deparaffinized in xylol. For enzymatic CD1a IHC staining, endogenous peroxidase was blocked using Methanol/0.3% H_2_O_2_ for 20 min, before slides were rehydrated in ethanol and demineralized in water baths. Antigen retrieval was performed in boiling citrate buffer (pH 6.0) for 10 min and sections were incubated overnight with mouse IgG1-anti-human CD1a antibody (Clone 010, 1:800, DAKO) diluted in phosphate buffered saline (PBS)/0.5% bovine serum albumin (BSA). The next day, Envision+ System-HRP labeled polymer anti-mouse (DAKO) was applied for 30 min and color development was attained using commercial DAB+ (DAKO) for 10 min in the dark. This reaction was stopped using demineralized water and slides were counterstained with Mayer's hematoxylin (Klinipath) for 5 s prior to mounting with Pertex (Leica Microsystems).

An earlier published protocol was used for triple CD1a/CD3/CD8 fluorescent IHC staining (14). In brief, antigen retrieval was performed in boiling EDTA buffer (pH 8.0) for 10 min followed by a blocking step using 10% Normal Goat Serum in PBS/0.5% BSA for 15 min at room temperature. Slides were incubated overnight with the following primary antibody mix: rabbit IgG-anti-human CD3 (polyclonal, 1:300, DAKO), mouse IgG2b-anti-human CD8 (clone 4B11, 1:100, Novocastra, via Leica Microsystems), and mouse IgG1-anti-human CD1a (Clone 010, 1:400, DAKO). The next day, tissue slides were incubated for 30 min in the dark with 1:300 diluted goat-anti-mouse IgG1 Alexa Fluor 488, goat-anti-mouse IgG2b Alexa Fluor 546 and goat-anti-mouse IgG2a Alexa Fluor 647 antibodies (all from Invitrogen, via Thermofisher Life Technologies Europe). After washing in PBS, the sections were mounted with Mowiol (homemade) or Prolong Gold (Thermo Fisher Scientific) and stored in the dark at 4°C.

### *BRAF*^*V600E*^ Mutation Analysis

CD1a^+^ enriched tissue parts were marked by a blinded pathologist on enzymatically CD1a stained LCH tissue slides. Based on these reference slides, CD1a^+^ enriched tissue parts were manually microdissected from multiple consecutively cut 10 μm tissue sections prepared from the remainder of the LCH tissue blocks. Total nucleic acid was automatically isolated from microdissected tissue using the Siemens Tissue Preparation System (Siemens Healthcare) robot (55). Presence of the *BRAF*^*V600E*^ mutation was assessed by allele-specific real-time qPCR, as previously described (56). Of the *n* = 54 *BRAF*^*V600E*^ negative samples, absence of the *BRAF*^*V600E*^ mutation was confirmed in 46 samples (85%) by next-generation sequencing (*n* = 39), whole exome sequencing (*n* = 1) (57) or *BRAF*^*V600E*^ droplet digital PCR (*n* = 6).

### Quantification of T Cell Density in LCH-Lesions

For the manual cell counting method, multiple representative images were taken of each tissue slide at 400× magnification using a conventional Leica DM5500 fluorescent microscope equipped with LAS AF software (Leica Microsystems). Images were solely taken of representative areas containing phenotypically aberrant CD1a^+^ LCH-cells. Using Image J software (version 1.47v) with the public Cell Counter plugin, fluorescently stained CD1a^+^, CD3^+^CD8^−^ and CD3^+^CD8^+^ cells were manually counted in all images by two independent researchers (PGK and ECS) who were unaware of patient identity and outcome data. The cell counts of the individual images were added to form total CD1a^+^, CD3^+^CD8^−^ and CD3^+^CD8^+^ cell counts. When total cell counts differed more than 10% between the two researchers, a third researcher (AGSH) reviewed the cell counting results and selected the most appropriate scoring (19/101 cases). Total CD3^+^ cell counts were obtained by adding total CD3^+^CD8^−^ and CD3^+^CD8^+^ cell counts. To adjust for substantial differences in biopsy size between different patients, which may lead to profound disparities in absolute numbers of counted cells, ratios between the final numbers of total CD3^+^ and CD3^+^CD8^+^ T cells and CD1a^+^ LCH-cells were calculated for each patient.

For the manual semi-quantitative eyeball estimation method, whole slide images were taken of the same immunostained tissue slides at 400× magnification using a Pannoramic 250 Flash II slidescanner (3DHISTECH). These images were scored semi-quantitatively for LCH-lesional CD3^+^ and CD3^+^CD8^+^ T cell density as has been previously described (58, 59): 1+, no, or sporadic T cells; 2+, moderate number of T cells; 3+, abundant occurrence of T cells; and 4+, highly abundant occurrence of T cells. Scoring examples are shown in Figure S1. Unfortunately, *n* = 21/101 (21%) of the tissue slides could not be reanalyzed due to considerable photobleaching of the fluorophores, induced by the earlier collection of high-power images for the manual cell counting analysis. Slides were scored independently by three researchers (PGK, ECS and AGSH). When scorings between two or more researchers differed more than 1 value (15/80 cases), the scoring was reviewed by all three researchers collectively and a consensus score was attained. Otherwise, the average score of the three scorings determined the final result, rounded to the nearest whole value (1–4+).

Whole slide images of sufficient quality (without significant color casts and/or folded tissue parts that are highly autofluorescent and/or out of focus) from *n* = 48 LCH-patients were analyzed using a quantitative automated digital image analysis method (Figure S2). First, the LCH-lesion and its directly adjacent T cells were encircled in the whole slide image in CaseViewer software and exported. In this way, cells that clearly did not belong to the microenvironment of the CD1a^+^ LCH-cells were excluded. Using a custom in-house developed macro in ImageJ software, a white balance was then set for each individual exported image by designating background, foreground and autofluorescence. Next, uniform color thresholds for green (CD1a^+^), red (CD3^+^CD8^−^), and purple (CD3^+^CD8^+^) were applied to all images, so that only green, red, and purple areas with color intensities higher than the threshold remained. Since automated quantification of individual cells was not feasible, the cumulative area of the remaining green, red, and purple areas was measured for each image, representing the total quantity of CD1a^+^, CD3^+^CD8^−^, and CD3^+^CD8^+^ cells. Purple and Red (CD3^+^) area/Green (CD1a^+^) area and Purple (CD3^+^CD8^+^) area/Green (CD1a^+^) area ratios could then be calculated for each patient. Comparison of the results obtained using our three separate analysis methods showed substantial concordance (Figure S3), supporting the validity of the findings in this study.

### *In vitro* Peptide-HLA Class I Binding Analysis

Competition-based peptide-HLA class I binding assays were performed as previously described (60). The HLA binding affinities of the target peptides and strong binding reference peptides are expressed as the concentration that inhibits 50% binding of a fluorescently-labeled standard peptide (IC_50_). The standard peptides were FLPSDCFPSV for HLA-A^*^02:01 and KVFPCALINK for HLA-A^*^03:01 and HLA-A^*^11:01. Notably, the ratio between the IC_50_ of a target peptide and the IC_50_ of an established strong binding reference peptide (for example 260:250 vs. 100:5) provides superior information on the true HLA class I binding capacity of the target peptide than the absolute IC_50_ of the target peptide.

### Generation of *BRAF*^*V600E*^ Expressing EBV-LCLs

The full length *BRAF*^*V600E*^ sequence incorporated in a pBABE-Puro-BRAF-V600E plasmid was re-cloned into a LZRS-ires-Green Fluorescent Protein (GFP) retroviral vector by introducing the SwaI restriction site and a kozak sequence in front of the ATG start codon at the 5′end of the *BRAF*^*V600E*^ sequence using Phusio DNA polymerase. In addition, a stop codon and NotI restriction site was introduced at the 3′end of the *BRAF*^*V600E*^ sequence. The original pBABE-Puro-BRAF-V600E plasmid was kindly provided by William Hahn (Addgene plasmid #15269; http://n2t.net/addgene:15269; RRID:Addgene_15269) (61). Ligation of the *BRAF* PCR product in the LZRS vector digested with SwaI and NotI was performed overnight at 16°C. Prior to spin inoculation of Phoenix packaging cells, the correct sequence of the re-cloned *BRAF*^*V600E*^ gene was confirmed by Sanger sequencing (data not shown). Retrovirus containing supernatant was subsequently used to transduce Epstein-Barr virus-immortalized B cell lines (EBV-LCLs) with either a control empty LZRS vector (mock transduced EBV-LCL) or with the new *BRAF*^*V600E*^ containing LZRS vector (*BRAF*^*V600E*^ transduced EBV-LCL). Stably transduced GFP^high^ cells were purified using an ARIA3 flow cytometer prior to bulk expansion in RPMI medium containing 10% bovine serum.

### Mass Spectrometry-Based Targeted Peptidomics

Cells were lysed at a concentration of 100e6 cells/ml lysis buffer [50 mM Tris-Cl pH 8.0, 150 mM NaCl, 5 mM EDTA, 0.5% Zwittergent 3–12 (N-dodecyl-N,N-dimethyl-3-ammonio-1-propanesulfonate) and protease inhibitor (Complete, Roche Applied Science)] for 2 h at 0°C (62). Lysates were successively centrifuged for 10 min at 2,500 × g and for 45 min at 31,000 × g to remove nuclei and other insoluble material, respectively. Next, lysates were cleared through a CL-4B Sepharose column (1 ml/1e9 cells) and passed through an anti-panHLA class I column containing 2.5 mg W6/32 IgG per ml protein A Sepharose (62). The W6/32 column was washed three times each with 1 ml of lysis buffer, 3 ml of low salt buffer (20 mM Tris-Cl pH 8.0, 120 mM NaCl), 1 ml of high salt buffer (20 mM Tris-Cl pH 8.0, 1 M NaCl), and finally with 3 ml of low salt buffer. Peptides were eluted with 5 ml of 10% acetic acid per ml column, diluted with 10 ml of 0.1% formic acid and purified by SPE (Oasis HLB, Waters) using 20 and 30% acetonitrile in 0.1% formic acid to elute the peptides.

For parallel reaction monitoring (PRM) analyses, the samples were lyophilized and resuspended in buffer A. HLA-eluates were injected together with a mix of 40 fmol of each heavy labeled peptide. The Orbitrap Fusion LUMOS mass spectrometer was operated in PRM-mode. Peptides KIGDFGLATE, KIGDFGLATV, KIGDFGLAT**E**K, and KIGDFGLATVK were monitored. Selected peptides, the transitions and collision energies can be found in Table S1. The isolation width of Q1 was 1.2 Da. MS2 resolution was 35,000 at an AGC target value of 1 million at a maximum fill time of 100 ms. The gradient was run from 2 to 36% solvent B (20/80/0.1 water/acetonitrile/formic acid (FA) v/v) in 120 min. The nano-HPLC column was drawn to a tip of ~5 μm and acted as the electrospray needle of the MS source. PRM data analysis and data integration were performed in Skyline 3.6.0.10493. Peptide abundances were calculated by comparing the peak area of the eluted (light) and the peak area of the spiked-in heavy peptides.

### Statistical Analysis

Statistical analysis was performed using GraphPad Prism version 8.0.1 and IBM SPSS Statistics version 25. Comparisons of (sub)groups were performed with the Mann-Whitney U test for continuous data and the Fisher exact test for categorical data. The Cox proportional hazards model was used for univariate analysis. Notably, log transformation of the widely differing CD8^+^ T cell:CD1a^+^ LCH-cell ratios was performed to increase the validity of the univariate analysis. Survival curves were estimated with the Kaplan-Meier method and compared with the Log-rank test. A *p*-value of <0.05 was considered statistically significant.

## Results

### LCH-Cells Express Normal Levels of HLA Class I and II Molecules at Their Cell Surface

**Figure 1:**
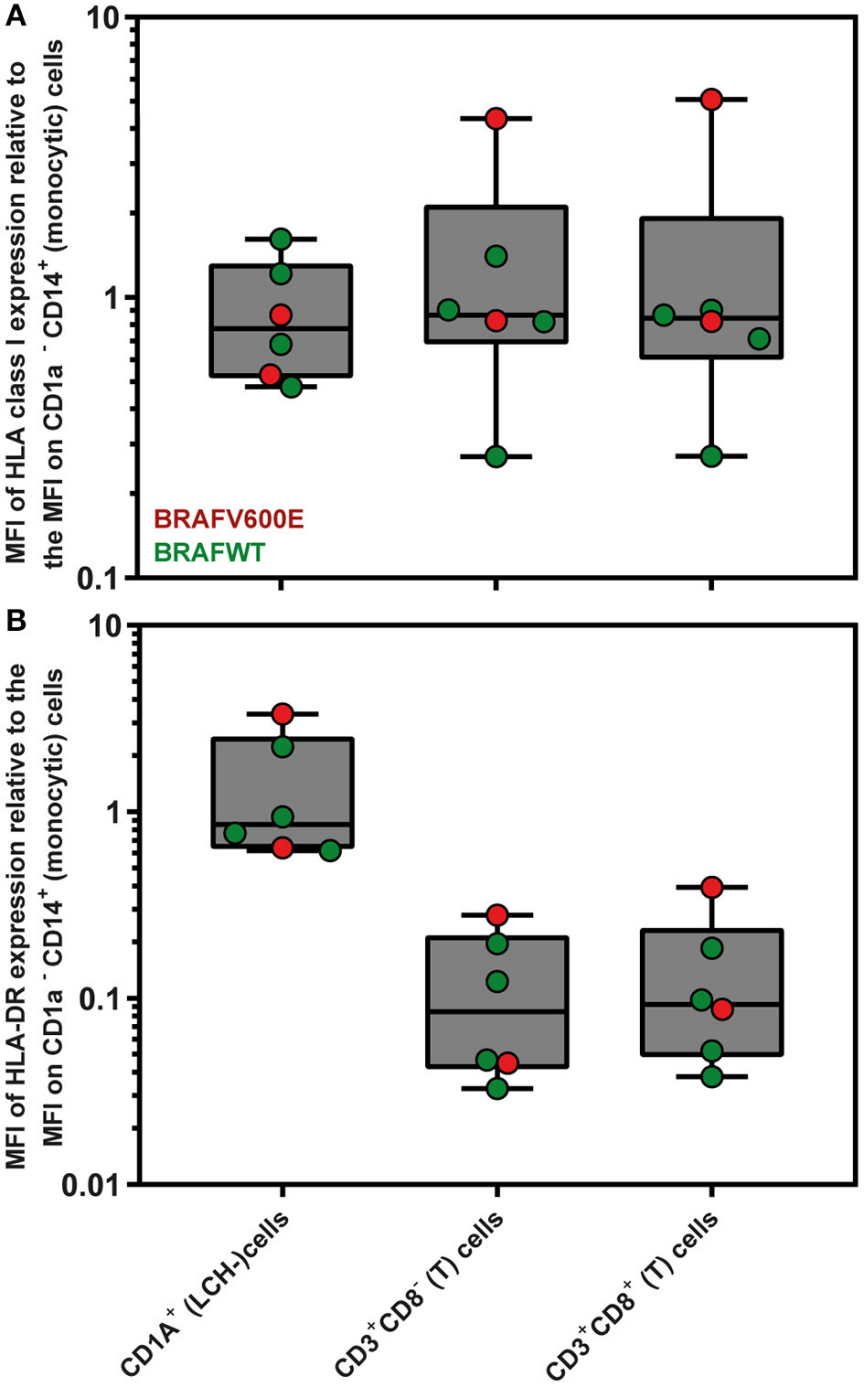
Flow cytometric measurements of HLA class I and HLA-DR expression by LCH biopsy-derived hematopoietic cells. Mean Fluorescent Intensity (MFI) of **(A)** HLA class I (W6/32) and **(B)** HLA-DR expression by live CD1a^+^ (LCH-)cells, CD3^+^CD8^−^ (T) cells and CD3^+^CD8^+^ (T) cells are depicted relative to the MFI of HLA class I or HLA-DR expression by CD1a^−^ CD14^+^ (monocytic) cells. The gating strategy applied is shown in Figure S4. The box extends from the 25th to 75th percentiles. The line in the middle of the box is plotted at the median. The whiskers go down to the smallest value and up to the largest. Each individual value is plotted as a point superimposed on the graph.

Since loss or downregulation of HLA expression has been shown to be a major tumor escape mechanism from T lymphocytes in a wide variety of cancers (63), we first evaluated by flow cytometric analysis the levels of HLA class I and HLA-DR expression on the surface of CD1a^+^ (LCH-)cells present in *n* = 6 LCH-biopsies. The gating strategy applied is shown in Figure S4. The mean fluorescent intensity (MFI) of HLA class I and HLA-DR expression by CD1a^+^ (LCH-)cells was comparable to MFI levels of HLA class I and HLA-DR expression by CD1a^−^ CD14^+^ (monocytic) cells present in the same LCH-biopsies (Figure 1; HLA class I, *p* = 0.69; HLA-DR, *p* = 0.94).

### The HLA Genotype of LCH-Patients Does Not Differ From Healthy Controls

Besides HLA expression, HLA subtype is a crucial factor influencing whether a (neo)antigen is actually presented at the surface of nucleated cells. Several earlier published studies have suggested associations between particular HLA subtypes and LCH disease (extension) (64–67). To investigate this, we compared HLA genotype data from *n* = 94 Dutch LCH-patients to the HLA genotypes of 5,604 healthy Dutch blood donors reflecting the HLA genotype of the Dutch population (50). To maintain sufficient statistical power, HLA genotype was compared at low resolution level. No significant differences between Dutch LCH-patients and the Dutch reference population were observed (Table S2). Thus, our data do not support previous reports describing excess frequency of HLA-Bw61 and HLA-Cw7 (64), HLA-B7 and HLA-DR2 (65), and HLA-DR4 and/or HLA-Cw7 (66) genotypes in LCH-patients (Tables S2, S3). Moreover, our results neither confirm that LCH-patients with unifocal bone disease have significantly more often HLA-DR4 and/or HLA-Cw7 (66) subtypes nor that patients with single-system LCH have an increased prevalence of HLA-DRB1^*^03 (67) when compared to patients with multisystem LCH (Table S4 and Figure S5, respectively).

### *BRAF*^*V600E*^ Mutation Correlates With Decreased CD8^+^ T Cell Density in LCH-Lesions

**Figure 2:**
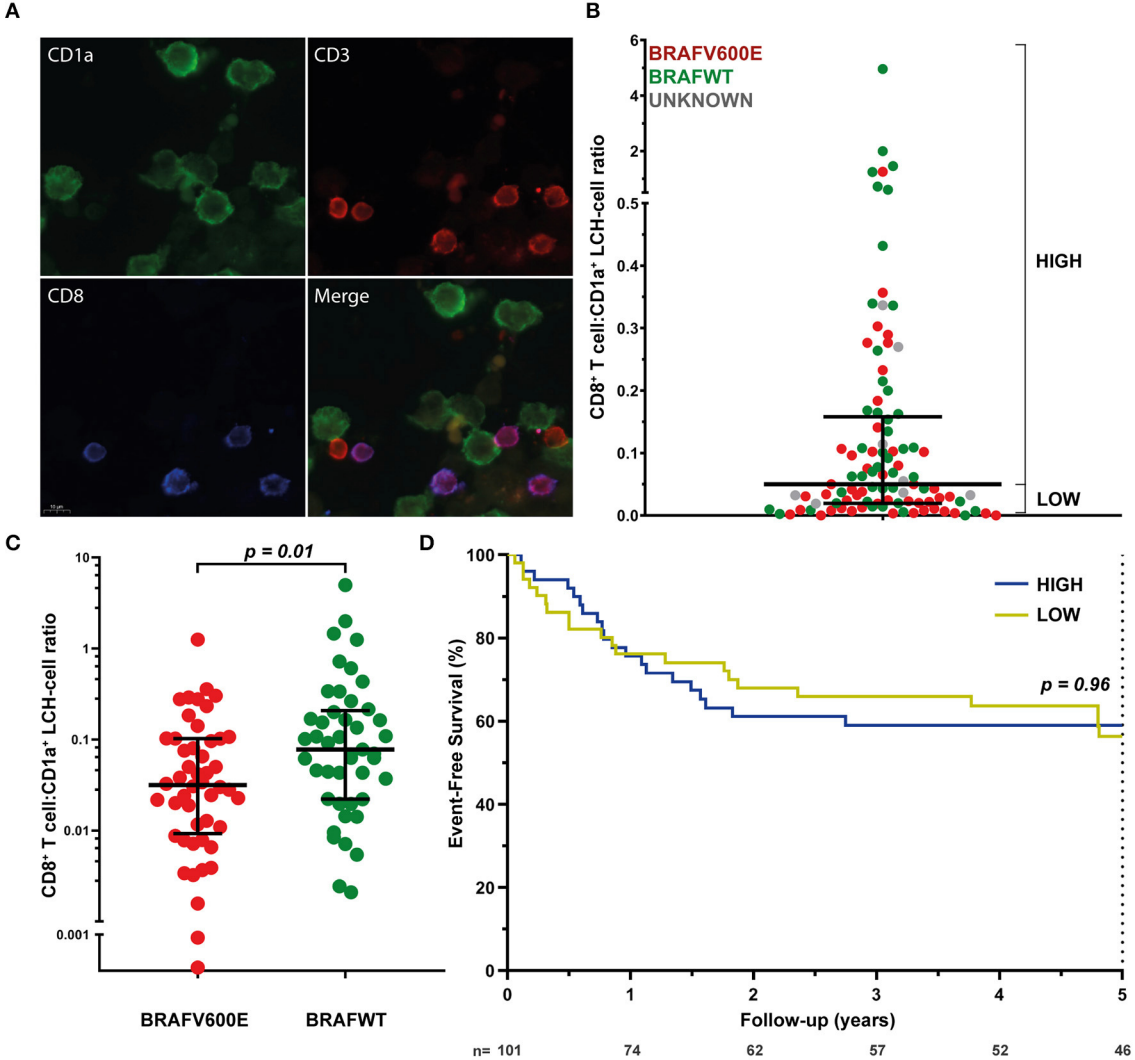
LCH-lesional CD8^+^ T cell densities in first disease onset tissue biopsies of LCH-patients. **(A)** Representative images of immunostained phenotypically aberrant CD1a^+^ LCH-cells (green), CD3^+^CD8^−^ T cells (red), and CD3^+^CD8^+^ T cells (purple) that were manually counted. **(B)** Distribution of LCH-lesional CD8^+^ T cell:CD1a^+^ LCH-cell ratios (CD8 ratios) in first-disease onset tissue biopsies of *n* = 101 LCH-patients. For Kaplan-Meier survival analysis (shown in **D**), patients were divided by a median split and grouped in patients with HIGH or LOW CD8 ratios. **(C)** Distribution of CD8 ratios in *BRAF*^*V600E*^ mutated (*n* = 48) and *BRAF* wildtype (*n* = 45) LCH-lesions. **(D)** Kaplan-Meier survival analysis of patients with HIGH (*n* = 50) or LOW (*n* = 51) CD8 ratios. Event was defined as LCH disease progression or reactivation. N, number of patients at risk.

Assured that LCH-cells express HLA class I (and II) molecules and that there is a normal HLA subtype distribution among LCH-patients, we next investigated the presence of CD8^+^ T cells in LCH-lesions. Various methods for the quantification of cell numbers in (specific areas of) tissue sections exist, including eyeball estimation, manual cell counting and automated digital image analysis. Although automated digital image analysis is increasingly being applied, manual cell counting is still considered the golden standard (68). Accordingly, we first determined the relative number of total CD3^+^ and CD3^+^CD8^+^ T cells in LCH-lesions using this method. Fluorescently stained CD1a^+^, CD3^+^CD8^−^ and CD3^+^CD8^+^ cells (Figure 2A) were manually counted in LCH-biopsies from *n* = 101 patients collected at first disease onset using the public ImageJ Cell Counter plugin. A median of 1,810 cells (range: 188–9,301) were counted in a median of 16 representative images (range: 2–56) taken at 400× magnification of tissue areas containing phenotypically aberrant CD1a^+^ LCH-cells. Large inter- and intrapatient heterogeneity was seen in the relative number of LCH-lesional CD3^+^ and CD8^+^ T lymphocytes (Figure S6 and Figure 2B, respectively). Calculated CD8^+^ T cell:CD1a^+^ LCH-cell ratios (CD8 ratios) ranged from 0.00 to 4.96. The median CD8 ratio was 0.06, corresponding to 1 CD8^+^ T cell per 16 CD1a^+^ LCH-cells. No significant difference in LCH-lesional CD8 ratios was observed between bone and skin biopsies (*p* = 0.37) nor between patients with single- or multisystem LCH disease (*p* = 0.55). Yet, *BRAF*^*V600E*^ mutated patients displayed significantly lower LCH-lesional CD8 ratios when compared to *BRAF* wildtype (*BRAF*^*WT*^) patients (*p* = 0.01; Figure 2C). *BRAF*^*V600E*^ mutated LCH-lesions had a median CD8 ratio of 0.0316, corresponding to 1 CD8^+^ T cell per 32 CD1a^+^ LCH-cells. In contrast, *BRAF*^*WT*^ lesions had a median CD8 ratio of 0.0775, corresponding to 1 CD8^+^ T cell per 13 CD1a^+^ LCH-cells. *BRAF*^*V600E*^ mutated lesions also had significantly lower total CD3^+^ T cell:CD1a^+^ LCH-cell ratios than *BRAF*^*WT*^ lesions (*p* = 0.001; Figure S7). As manual selection of representative tissue areas may introduce bias, we also analyzed whole slide images taken from a subset of immunostained tissue sections using a previously described semi-quantitative eyeball estimation method (58, 59) (Figure S1) and a quantitative automated digital image analysis method (Figure S2). The correlation between the *BRAF*^*V600E*^ mutation and decreased LCH-lesional CD3^+^ and CD8^+^ T cell density was confirmed by these two additional analysis methods (Figure S8).

### Lesional CD8^+^ T Cell Density Does Not Correlate With Event-Free Survival in LCH

We subsequently assessed whether lesional CD8^+^ T cell density is of prognostic value in LCH. Using univariate cox regression analysis, no significant association was observed between LCH-lesional CD8 ratio and event-free survival (*p* = 0.46; Hazard Ratio = 0.89; 95% Confidence Interval = 0.66–1.21). In addition, no significant difference was present when patients were divided by a median split, grouped in patients with HIGH or LOW CD8 ratios (Figure 2B and Table 1) and compared with regard to event-free survival (*p* = 0.96, Figure 2D). Thus, LCH-lesional CD8^+^ T cell density did not correlate with disease outcome in this retrospective patient cohort.

**Table 1:** Characteristics of LCH-patients from whom biopsies were analyzed for LCH-lesional CD8^+^ T cell density.

	**All patients**	**High LCH-lesional CD8 ratio**	**Low LCH-lesional CD8 ratio**	***P*-value**
Patients	101	50 (50%)	51 (50%)	
**Gender**				
Male	53 (52%)	29 (58%)	24 (47%)	0.32
Female	48 (48%)	21 (42%)	27 (53%)	
**Age distribution**				
Pediatric patients	85 (84%)	40 (80%)	45 (88%)	0.29
Adult patients	16 (16%)	10 (10%)	6 (12%)	
**Disease extension**				
SS	78 (77%)	39 (78%)	39 (76%)	1
MS RO–	12 (12%)	5 (10%)	7 (14%)	0.76
MS RO+	11 (11%)	6 (12%)	5 (10%)	0.76
**Mutation status**				
*BRAF^*V600E*^* positive	48 (48%)	18 (36%)	30 (59%)	0.02
*BRAF^*V600E*^* negative	45 (45%)	28 (56%)	17 (33%)	
Unknown	8 (8%)	4 (8%)	4 (8%)	
**Chemotherapy for FDO**	34 (34%)	15 (30%)	19 (37%)	0.53
**Follow-up (median)**	10.1 years	8.5 years	11.3 years	0.36

*SS, single-system LCH disease; MS RO–, multisystem LCH disease without risk organ (bone marrow, liver and/or spleen) involvement; MS RO+, multisystem LCH disease with risk organ involvement; FDO, first disease onset; CD8 ratio, CD8^+^ T cell:CD1a^+^ LCH-cell ratio*.

### The *BRAF*^*V600E*^ Derived Neopeptide KIGDFGLATEK Binds to HLA-A^*^03:01 and HLA-A^*^11:01

To investigate the immunogenicity of the *BRAF*^*V600E*^ mutation, we used the online NetMHC 4.0 server (69) to explore putative HLA class I binding 8–12 amino acid long (8–12mer) neopeptides derived from the *BRAF*^*V600E*^ protein. In addition, NetCHOP 3.1 software (70) was used to predict proteasomal cleavage motifs and thereby identify peptides that are presumably generated by the human proteasome. From all 8–12mer *BRAF*^*V600E*^ derived neopeptides that are generated by the human proteasome according to NetCHOP, only a single neopeptide, the 11mer KIGDFGLAT**E**K, is predicted to bind to one or more of the analyzed HLA class I molecules (Table S5). According to NetMHC, KIGDFGLAT**E**K binds weakly to HLA-A^*^11:01 and HLA-A^*^03:01, expressed by respectively *n* = 11/104 (11%) and *n* = 25/104 (24%) LCH-patients from our cohort. The remainder of the 8–12mer *BRAF*^*V600E*^ derived neopeptides are all considered not be generated by the human proteasome and/or to be non-binders. Additional *in vitro* peptide-HLA binding studies however demonstrated that KIGDFGLAT**E**K binds with comparable affinity to HLA-A^*^11:01 as the strong binding reference peptide (QVPLRPMTYK) that was used in our competition-based peptide-HLA binding assay (60). In line with the predicted binding affinity, this neopeptide was shown to also bind, albeit less efficiently, to HLA-A^*^03:01 (Table 2), as evidenced by the small difference in nanomolar concentration that inhibited 50% binding (IC_50_) of the fluorescently-labeled standard peptide (KVFPCALINK) between KIGDFGLAT**E**K and QVPLRPMTYK (672 vs. 297 nM, respectively). Notably, NetMHCstab 1.0 software (71) predicts that the KIGDFGLAT**E**K-HLA-A^*^11:01 complex is highly stable (predicted half-life: 8.87 h) and that the KIGDFGLAT**E**K-HLA-A^*^03:01 complex is weakly stable (predicted half-life: 3.29 h). We also assessed the *in vitro* HLA binding affinity of the 11mer KIGDFGLATVK and 10mer KIGDFGLATV *BRAF* wildtype peptides and of the 10mer KIGDFGLATE neopeptide (Table 2). In accordance with the predictions made by NetMHC, KIGDFGLATVK was shown to bind with comparable affinity to HLA-A^*^11:01 as the strong binding reference peptide QVPLRPMTYK, and to confer weaker binding to HLA-A^*^03:01, just like KIGDFGLAT**E**K. Moreover, the 10mer *BRAF* wildtype peptide KIGDFGLATV was shown to bind with comparable affinity to HLA-A^*^02:01 as the strong binding reference peptide (FLPSDFFPSV) used in our assay. In contrast, its mutant counterpart KIGDFGLATE does not bind at all to this particular HLA class I molecule.

**Table 2:** *In silico* and *in vitro* HLA class I binding affinities of *BRAF*^*V600E*^ and *BRAF* wildtype protein-derived peptides and two strong binding reference peptides.

**Peptide**	**Predicted proteasomal cleavage**	**Predicted HLA binding affinity (IC**_****50****_**, nM)**						***In vitro*** **HLA binding affinity (IC**_****50****_**, nM)**			**500 nM**
**NetMHC 3.4**			**NetMHC 4.0**			**Peptide-HLA binding assay**					
**NetCHOP 3.1**	A*02:01	A*03:01	A*11:01	A*02:01	A*03:01	A*11:01	A*02:01	A*03:01	A*11:01		
**KIGDFGLAT**V	YES	38	17,310	23,823	107	13,515	19,725	40	NT	NT	**50 nM**
**KIGDFGLAT**$\underline{\text{E}}$	NO	15,997	20,112	23,609	19,485	22,113	23,743	86918	NT	NT	
**KIGDFGLAT**V**K**	YES	25,719	448	163	3,347	191	363	NT	415	32	
**KIGDFGLAT**$\underline{\text{E}}$**K**	YES	23,298	622	98	29,947	278	345	NT	672	45	
**QVPLRPMTYK**	NR	31,545	77	62	32,523	21	37	NT	297	36	
**FLPSDFFPSV**	NR	4	24,267	27,281	4	19,261	22,553	8	NT	NT	**0 nM**

*IC_50_, the concentration that inhibits 50% binding of a fluorescently-labeled standard peptide; nM, nanomolar; NR, not relevant, because these are the strong binding reference peptides; NT, not tested. The color values correspond to the IC_50_ values 0–500 nM (0 dark red, 500 white)*.

### HLA-A^*^11:01 and/or HLA-A^*^03:01 Genotype Is Not Associated With Increased Event-Free Survival in *BRAF*^*V600E*^ Mutated LCH-Patients

Having established that the *BRAF*^*V600E*^ derived neopeptide KIGDFGLAT**E**K can bind to two HLA class I molecules that are relatively frequent in the Caucasian population, we evaluated whether *BRAF*^*V600E*^ mutated LCH-patients expressing HLA-A^*^03:01 and/or HLA-A^*^11:01 had increased event-free survival as compared to LCH-patients without these HLA genotypes. High-resolution HLA genotype data was available for *n* = 48 *BRAF*^*V600E*^ mutated LCH-patients. Patient characteristics are shown in Table S6. No significant difference in event-free survival was observed between *BRAF*^*V600E*^ mutated LCH-patients with and without HLA-A^*^03:01 and/or HLA-A^*^11:01 (*p* = 0.32, Figure S9).

### KIGDFGLATEK Is Not Detected in the HLA Class I Peptidome of *BRAF*^*V600E*^ Expressing Cells

To assess whether KIGDFGLAT**E**K is actually presented on the surface of cells that express *BRAF*^*V600E*^ and HLA-A^*^03:01 and/or HLA-A^*^11:01, we performed mass spectrometry-based targeted peptidomics of HLA class I presented peptides isolated from various EBV-LCL transduced with a LZRS-retroviral vector containing full length *BRAF*^*V600E*^ protein and reporter Green Fluorescent Protein (GFP) encoding DNA sequences. Based on the results of the *in silico* analysis and *in vitro* peptide-HLA binding assays, three different EBV-LCL were selected for the transduction experiments with HLA-A^*^03:01/HLA-A^*^02:01 (SB), HLA-A^*^11:01/HLA-A^*^02:01 (MLA), and HLA-A^*^02:01/HLA-A^*^02:01 (JY) genotypes. Extended HLA genotypes are shown in Table S7. After retroviral transduction, GFP^high^ cells were sorted and expanded in bulk. JY and MLA cell lines that were mock transduced with a control (empty-)GFP retroviral vector were analyzed in parallel. Flow cytometric analysis demonstrated that neither retroviral transduction with the *BRAF*^*V600E*^ containing vector (Figure S10) nor transduction with the control empty vector (data not shown) altered HLA class I (W6/32) and HLA-DR expression at the cell surface. Moreover, HLA subtype-specific antibodies (kindly provided by Dr. D.L. Roelen, HLA genotyping laboratory LUMC, Leiden) confirmed normal HLA subtype expression by *BRAF*^*V600E*^ transduced SB, MLA (Figure S10) and JY cells (data not shown). We also included an HLA-A^*^01/HLA-A^*^24 bearing *BRAF*^*V600E*^ mutated colon carcinoma cell line (HT29) with earlier confirmed HLA (72–74) and *BRAF*^*V600E*^ protein (75, 76) expression in our analysis. Using parallel reaction monitoring (PRM)-based targeted peptidomics (77), the 11mer neopeptide KIGDFGLAT**E**K was not detected in the HLA class I peptidomes of both *BRAF*^*V600E*^ expressing cell lines expressing HLA-A^*^03:01 or HLA-A^*^11:01 (Table 3). Notably, neither the 11mer *BRAF* wildtype peptide KIGDFGLATVK was detected in HLA class I peptides isolated from the mock or *BRAF*^*V600E*^ transduced SB and MLA EBV-LCL. In contrast, the 10mer BRAF wildtype peptide KIGDFGLATV was detected in the HLA class I peptidomes of 3/3 *BRAF*^*V600E*^ transduced and 2/2 mock transduced cell lines expressing HLA-A^*^02:01 (Table 3).

**Table 3:** Peptides detected using mass-spectrometry based targeted peptidomics of HLA class I peptides isolated from multiple *BRAF* wildtype or *BRAF*^*V600E*^ expressing cell lines.

**Peptide**	**Cell line**											
	**JY mock**		**JY BRAF**^****V600E****^		**MLA mock**		**MLA BRAF**^****V600E****^		**SB BRAF**^****V600E****^		**HT29**	
	**1,900** **×** 10e6^*^		**51** **×** **10e6**		**158** **×** **10e6**		**170** **×** **10e6**		**28** **×** **10e6**		**1,800** **×** **10e6**	
	A^*^02:01	A^*^02:01	A^*^02:01	A^*^02:01	A^*^02:01	A^*^11:01	A^*^02:01	A^*^11:01	A^*^02:01	A^*^03:01	A^*^01	A^*^24
**KIGDFGLAT**V	+		+		+		+		+		–	
**KIGDFGLAT**$\underline{\text{E}}$	–		–		–		–		–		–	
**KIGDFGLAT**V**K**	–		–		–		–		–		–	
**KIGDFGLAT**$\underline{\text{E}}$**K**	–		–		–		–		–		–	

Mock, transduced with a control (empty-) GFP retroviral vector; BRAF^V600E^, transduced with a BRAF^V600E^-GFP retroviral vector; HT29, colon carcinoma cell line harboring the heterozygous BRAF^V600E^ mutation; +, peptide detected; –, peptide not detected;

*, *number of cells analyzed*.

## Discussion

A large number of studies have demonstrated a positive association between overall CD8^+^ T cell density in the tumor infiltrate and a favorable clinical prognosis in many different types of cancers (6). In this study, we did not observe such an association in a substantial cohort of LCH-patients with well-documented clinical outcome. This dissimilarity between LCH and other neoplastic disorders may be due to their vast differences in mutational load and, correspondingly, the number of T cell activating neoantigens that can arise from this mutational burden. Furthermore, the immune suppressive microenvironment in LCH-lesions (5, 14, 15, 18, 78–81) may hamper CD8^+^ T cell infiltration (non-mutated), antigen recognition and cytolytic function.

In line with an earlier undetailed observation (7), the relative number of LCH-lesional CD8^+^ T cells appears low in this study. Moreover, we demonstrate with three separate analysis methods that *BRAF*^*V600E*^ mutated LCH-patients display lower lesional CD3^+^ and CD8^+^ T cell densities than *BRAF* wildtype patients. Although the clinical significance of this latter observation is not immediately apparent, it does point out that the different MAPK pathway mutations expressed by neoplastic LCH-cells seem to have a distinct impact on their immune microenvironment. A number of studies on *BRAF*^*V600E*^ positive melanoma have already suggested that the *BRAF*^*V600E*^ mutation promotes immune evasion by upregulating the transcription of many immunomodulatory chemokine and cytokine genes as well as the internalization of cell surface HLA class I molecules (82, 83). The presence of many of these immunomodulatory chemokines and cytokines in LCH-lesions has been extensively demonstrated (2). Notably, we did however observe normal HLA class I expression by CD1a^+^ (LCH-)cells in two *BRAF*^*V600E*^ positive LCH-biopsies analyzed by flow cytometry (Figure 1A), and showed that transduction of EBV-immortalized B cells with a *BRAF*^*V600E*^ encoding retroviral vector does not impair HLA class I expression. Zeng and colleagues recently described that *BRAF*^*V600E*^ mutated LCH-patients have significantly higher numbers of lesional Foxp3^+^ regulatory T cells and increased PD-L1 expression by LCH-cells when compared to *BRAF*^*WT*^ patients (80). In accordance with this study, a preliminary report by Chakraborty and others also describes that *BRAF*^*V600E*^ expressing LCH-cells display higher expression levels of ligands for inhibitory receptors, including PD-L1/L2 and Galectin-9, when compared to *BRAF*^*WT*^ patients (19). Notably, the presence of PD-1 expressing T cells in LCH-lesions has been reported as well (18, 19), and was confirmed in (*BRAF*^*V600E*^ positive) patients from our cohort (Figure S11). PD-L1 blockade has been shown to induce expansion of tumor-infiltrating CD8^+^ T cells (84). Thus, the reported increased PD-L1 expression by *BRAF*^*V600E*^ positive LCH-cells (19, 80) could explain the decreased LCH-lesional CD8^+^ T cell density in *BRAF*^*V600E*^ mutated patients from our study. In addition, the immune suppressive microenvironment in LCH-lesions (5, 14, 15, 18, 78–81) may clarify why the rare CD8^+^ T cells that did make it into these lesions had no significant clinical impact. This is supported by our own observation of low numbers of HLA-DR^pos^ LCH-lesional CD8^+^ T cells (Figure 1), low numbers of “licensed-to-kill” CD8^+^ T cells co-expressing the cytolytic enzymes Perforin and Granzyme B (85) (Figure S12), and rare presence of Caspase 3 expressing LCH-cells (data not shown). HLA-DR is widely recognized as a marker of T cell activation (86), and Caspase 3 is the hallmark marker of efficient target cell apoptosis induced by activated CD8^+^ T cells (87). In line with the recently reported defective response of LCH-lesion infiltrating T cells to allogeneic stimulation (19), these observations collectively suggest that CD8^+^ T cells in LCH-lesions are often dysfunctional. Future studies using (imaging) mass cytometry, which allows the simultaneous detection of a multitude of cellular markers (with spatial context), are needed to study the phenotypic characteristics of LCH-lesional (CD8^+^) T cells in more detail. Moreover, the alleged distinct impact of the different MAPK pathway mutations on the immune microenvironment of neoplastic LCH-cells should ideally be investigated in a LCH mouse model.

Encouraged by published results suggesting that *BRAF*^*V600E*^ protein-derived neopeptides can trigger antitumor immunity (41, 82), we used the most recent version of publicly accessible NetMHC software to explore putatively HLA class I binding neoantigens derived from the *BRAF*^*V600E*^ protein. Surprisingly, from all 8–12mer *BRAF*^V600E^ derived neopeptides that are predicted to be generated by the human proteasome by NetCHOP 3.1 software, only a single neopeptide (KIGDFGLAT**E**K) was predicted to bind to one or more of the analyzed HLA class I molecules. *In vitro* peptide-HLA binding assays confirmed the predicted binding capacity of KIGDFGLAT**E**K to HLA-A^*^03:01 and HLA-11^*^01. In contrast to the results generated with an earlier version of Syphpeiti software (41), the NetMHC 4.0 server did not qualify the two (putatively HLA-A^*^02:01 binding) neopeptides LATEKSRWSG and LATEKSRWS to be HLA-binders. Using PRM-based targeted peptidomics, KIGDFGLAT**E**K was not detected in the HLA class I peptidomes of 2/2 *BRAF*^*V600E*^ expressing EBV-LCL (MLA *BRAF*^*V600E*^ and SB *BRAF*^*V600E*^) that expressed normal levels of HLA-A^*^03:01 or HLA-A^*^11:01. In contrast, the HLA-A^*^02:01 binding *BRAF* wildtype peptide KIGDFGLATV was traceable in HLA class I peptides isolated from 5/5 cell lines expressing this HLA subtype, verifying normal antigen processing in these cells and adequate sensitivity of our peptidomics approach. Since the 11mer *BRAF* wildtype peptide KIGDFGLATVK was not detected in mock (empty-GFP) nor *BRAF*^*V600E*^ transduced EBV-LCL as well, the apparent lack of KIGDFGLAT**E**K presentation at the cell surface seems not due to a competitive HLA binding disadvantage relative to its wildtype counterpart (88). Instead, both KIGDFGLAT**E**K and KIGDFGLATVK peptides may not be generated by the human proteasome. This could be explained by the fact that both HLA-A^*^03:01 and HLA-A^*^11:01 molecules exclusively bind peptides with lysine as the C-terminal anchor residue (89). NetCHOP software only produces neural network predictions for proteosomal cleavage. Protein cleavage yielding C-terminal lysine residues is, however, not readily accomplished by the human proteasomes alone. Instead, this process requires the cytosolic endopeptidases nardilysin and thimet oligopeptidase as well (89, 90). Another possibility is that the 11mer KIGDFGLATVK and KIGDFGLAT**E**K peptides are expressed at the cell surface, but that they are underrepresented among the large pool of naturally presented ligands eluted from peptide-HLA class I complexes, because of a common peptide length distribution including mostly 9mer peptides and far less 8mer, 10mer, and longer peptides (91). This is also demonstrated by the list of peptides that were detected using data-dependent acquisition-based peptidomics in the HLA class I peptide pools isolated from the mock transduced JY and MLA EBV-LCL (Table S8). The high sensitivity of targeted peptidomics makes this option however less probable, although it must be noted that lower numbers (28–170 × 10e6) of *BRAF*^*V600E*^ transduced SB, MLA, and JY B cells were subjected to analysis as compared to mock transduced B cells (158–1,900 × 10e6). This was because GFP^high^ *BRAF*^*V600E*^ transduced cells displayed intrinsically higher apoptosis rates leading to substantially lower yields (data not shown).

In addition to the importance of CD8^+^ T cells, multiple studies have highlighted the importance of CD4^+^ T cells in tumor rejection (34, 92–96). Notably, one study identified *BRAF*^*V600E*^-specific CD4^+^ T cells after repetitive peptide stimulation of peripheral blood mononuclear cells from three melanoma patients whose metastatic tumors harbored the *BRAF*^*V600E*^ mutation (40). Moreover, Veatch and colleagues recently identified HLA-DQB1^*^03-restricted *BRAF*^*V600E*^-specific CD4^+^ T cells in an acral melanoma patient, who nonetheless developed metastases under ipilimumab (anti-CTLA-4) immunotherapy (97). Unfortunately, the precise amino acid sequence of the recognized neoantigen was not reported. Available software tools to predict HLA class II binding peptides are known to be significantly less accurate than available algorithms for predicting HLA class I binding peptides. Moreover, the yield of *BRAF*^*V600E*^ transduced B cells expressing HLA-DQB1^*^03:02 (SB EBV-LCL) was far too small to elute sufficient quantities of peptide-HLA class II complexes needed for successful data-dependent acquisition-based peptidomics. We could, therefore, not confirm that this recently identified *BRAF*^*V600E*^ protein-derived HLA-DQB1^*^03 binding neopeptide is naturally processed and presented at the cell surface of our *BRAF*^*V600E*^ transduced HLA-DQB1^*^03 expressing EBV-LCL. We did however investigate whether *BRAF*^*V600E*^ mutated LCH-patients expressing HLA-DQB1^*^03 in general, or HLA-DQB1^*^03:02 and/or HLA-DQB1^*^03:03 in particular [due to their putative strongest binding and/or peptide-HLA complex stability (97)], had increased event-free survival when compared to *BRAF*^*V600E*^ mutated patients without these HLA genotypes. Notably, HLA class I subtype has already been demonstrated to influence response to checkpoint blockade immunotherapy in patients with diverse cancers (98). Neither *BRAF*^*V600E*^ mutated LCH-patients with HLA-DQB1^*^03 (*n* = 30, 62.5%) nor with HLA-DQB1^*^03:02 and/or HLA-DQB1^*^03:03 (*n* = 18, 37.5%) displayed increased event-free survival when compared to patients without these HLA alleles (*p* = 0.78 and *p* = 0.57, respectively; data not shown). Thus, although we agree that adoptive cell therapy with T cell receptor-engineered *BRAF*^*V600E*^-specific CD4^+^ T cells may offer great therapeutic potential, the clinical impact of potentially present *BRAF*^*V600E*^-specific CD4^+^ T cells in HLA-DQB1^*^03 bearing, *BRAF*^*V600E*^ mutated LCH-patients is questionable. Of note, the rare CD4^+^ *BRAF*^*V600E*^-specific T cells reported in the acral melanoma patient by Veatch et al. were not paralleled by *BRAF*^*V600E*^-specific CD8^+^ T cells, but by diverse CD8^+^ T cells reactive to multiple melanoma-associated self-antigens. Whether such non-mutated tumor-associated antigens are also expressed by LCH-cells is of great interest and remains to be determined. This will however be challenging given the (relatively) low numbers of neoplastic LCH-cells that can be obtained for peptidome analysis from fresh or frozen LCH tissue samples, which are in addition very scarce due to the rarity of the disease.

Since the generation of neoantigens is a probabilistic process (47), we can of course not rule out that other somatic mutations in LCH-cells are a source of neoantigens that are naturally processed and presented in (stable) peptide-HLA class I complexes. Based on recent insights, this probability is however very low. With the development of deep-sequencing technologies, comprehensive analyses of neoantigen-specific T cell responses have been carried out for a substantial number of cancer patients since 2013 (25, 26, 29). The striking conclusion that can now be drawn from these studies is that only a very small fraction of non-synonymous mutations leads to the formation of a neoantigen for which CD4^+^ or CD8^+^ T cell reactivity can be detected (25). Most melanomas and a sizable fraction of other high-prevalence cancers in adults have a mutational load above 10 somatic mutations per Mb, corresponding to ~150 non-synonymous mutations within expressed genes (25, 99, 100). Even in melanoma patients, neoantigen-specific T cell reactivity is however not always observed (95). Thus, there is a growing awareness that tumor types with a mutational load below 10, and especially below 1 mutation(s) per Mb, are less likely to express neoantigens that can be recognized by autologous T cells (25). Although the total number of LCH samples analyzed by whole-exome sequencing (WES) is still small (101), a remarkably low frequency of somatic mutations in LCH-cells was found in the largest WES analysis to date (*n* = 41), with a median of 1 somatic mutation per patient (0.03 mutations per Mb) (22). Thus, the likelihood of neoantigen formation and concurrent induction of protective neoantigen-specific T cell responses in LCH-patients seems very low (25). Notably, Goyal and others recently demonstrated a low mutational burden in other histiocytic neoplasms as well (102). We therefore question the usefulness of classical immune checkpoint inhibitors for the treatment of relapsed or refractory LCH (or other histiocytic neoplasms), especially given that these LCH-patients often bear the *BRAF*^*V600E*^ mutation (44), and that pretherapy intratumoral CD8^+^ T cell density has been shown to positively correlate with mutational burden, neoantigen load and response to immune checkpoint inhibition in many other neoplastic diseases (103, 104).

## Data Availability Statement

The raw data supporting the conclusions of this article will be made available by the authors, without undue reservation, to any qualified researcher.

## Ethics Statement

The studies involving human participants were reviewed and approved by the Medical Ethical Committee of the LUMC. Written informed consent to participate in this study was provided by the participants or by the participants' legal guardian.

## Author Contributions

PK, ES, AH, QA, JB, SV, AR, GJ, PV, GH, KF, RE, NS-W, and TW performed experiments and/or analyzed data. DR performed low-resolution HLA genotyping and provided HLA subtype-specific antibodies. LV helped with the automated digital image analysis. RV, CN, AC, and CH provided LCH tissue biopsies and marked CD1a^+^ enriched tissue parts on CD1a stained slides that served as reference slides for the manual microdissection procedure. AB, JL, OA, and CB included patients and supervised the clinical data collection by PK, TZ, VL, and RME. AH, CB, and OA designed the study. PK and AH drafted the manuscript.

### Conflict of Interest

The authors declare that the research was conducted in the absence of any commercial or financial relationships that could be construed as a potential conflict of interest.
