# Supplementary material for: Apparent Lack of BRAFV600E Derived HLA Class I Presented Neoantigens Hampers Neoplastic Cell Targeting by CD8+ T Cells in Langerhans Cell Histiocytosis
Source: Front Immunol. 2020 Jan 10;10:3045. doi: 10.3389/fimmu.2019.03045 (PMC6967030; doi:10.3389/fimmu.2019.03045)
Supplement: Supplementary file 1 [file Data_Sheet_1.pdf]

SUPPLEMENTARY FIGURES

Figure S1. Representative scoring examples of LCH-lesional CD8⁺ T cell density using the semi-quantitative eyeball estimation method. In images depicted by an asterisk, tissue areas with considerable numbers of CD1a⁺ LCH-cells are encircled with dashed lines, and tissue areas with high CD8⁺ T cell density are depicted in purple.

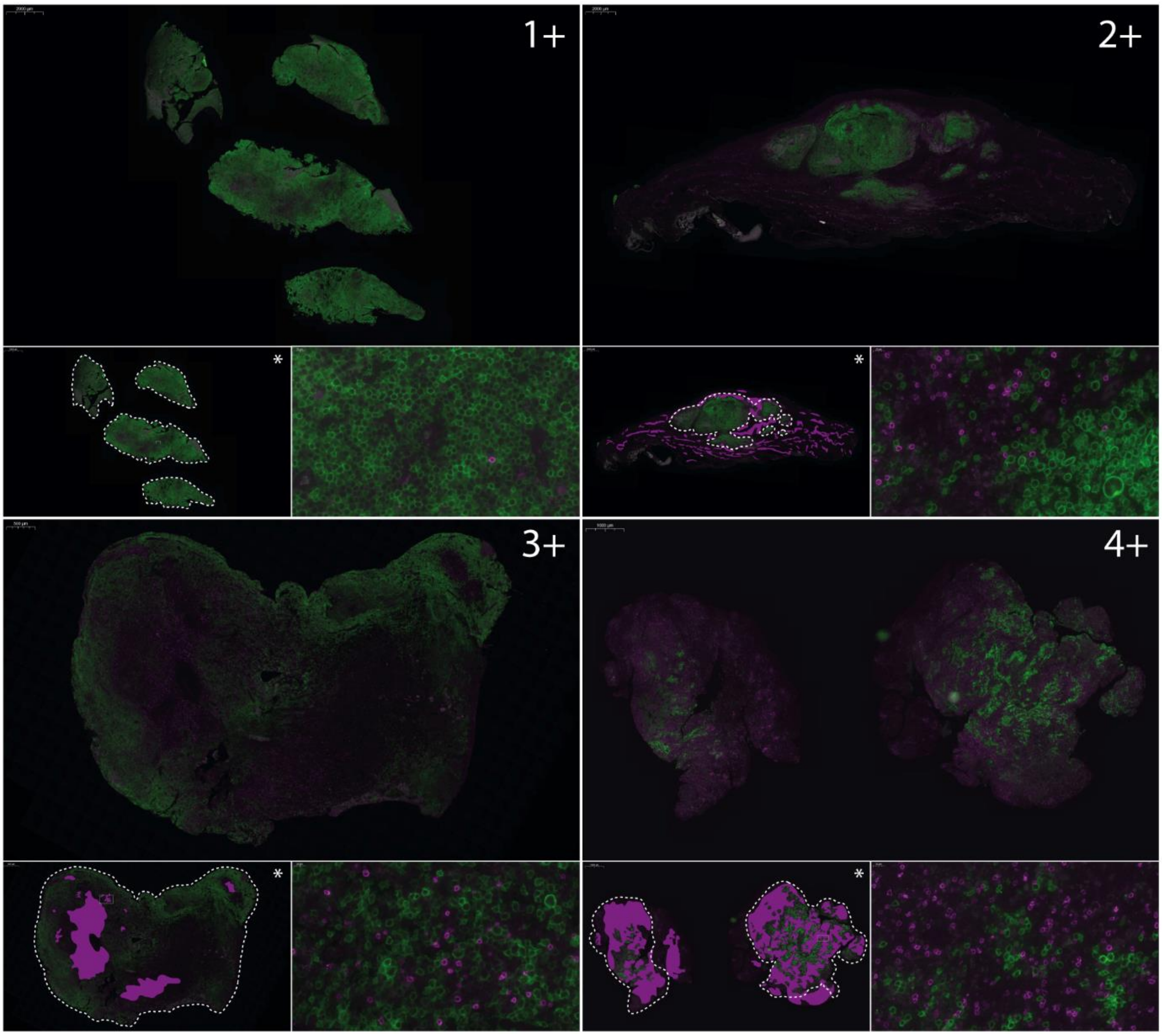

Figure S2. Flowchart of the automated digital image analysis method that was used for quantifying LCH-lesional $CD3^{+}CD8^{-}$ and $CD3^{+}CD8^{+}$ T cell density.

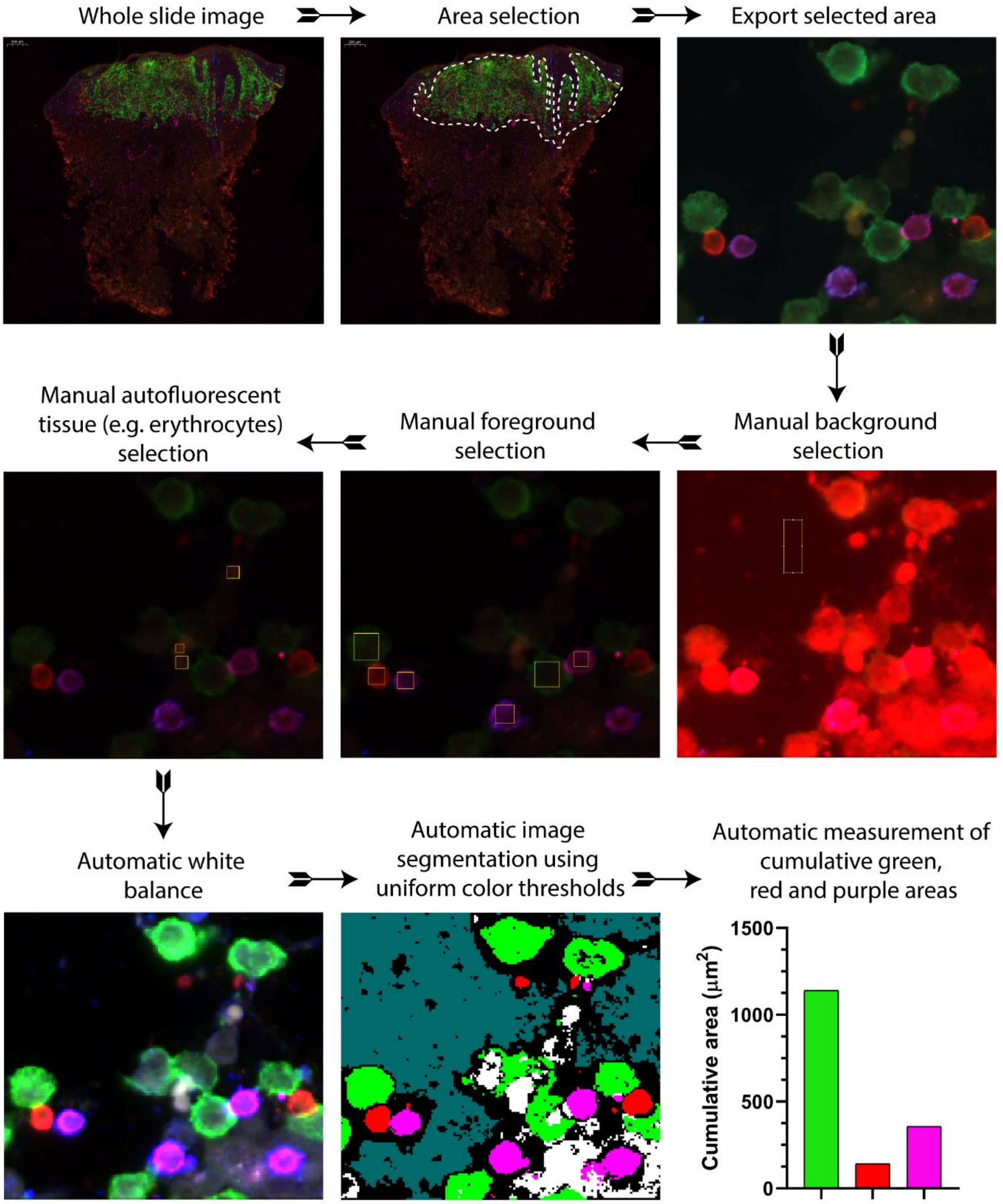

Figure S3. Comparison of the results obtained using the manual cell counting method, manual eyeball estimation method and automated digital image analysis method for quantifying LCH-lesional CD3^{+} or CD3^{+}CD8^{+} T cell density.

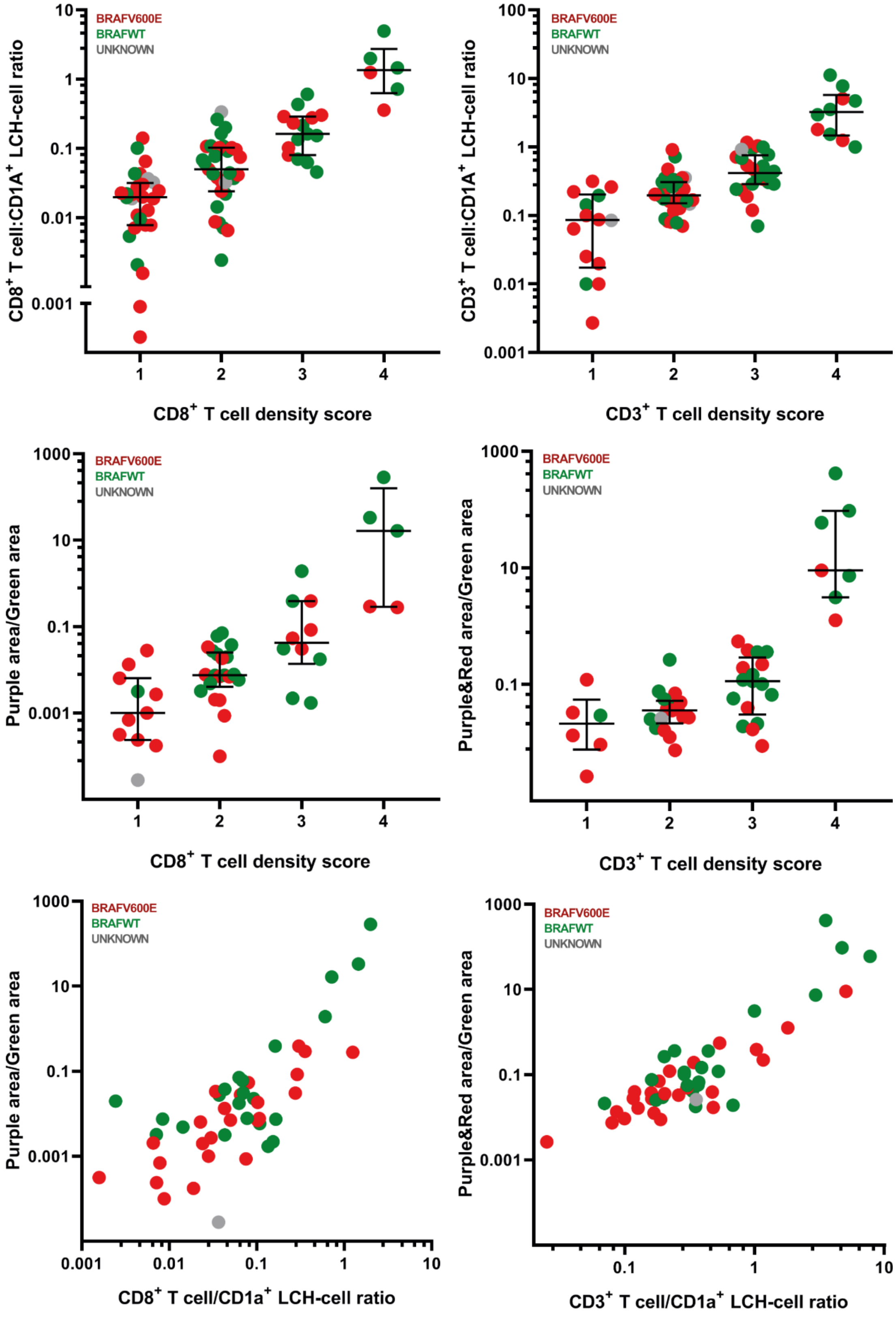

Figure S4. Gating strategy for multicolor flow cytometric analysis of HLA class I and HLA-DR expression on different types of LCH biopsy-derived cells. Representative FACS dot-plots and corresponding histograms are shown. Positive staining was determined in relation to the fluorescent intensity of unstained cells tested in parallel.

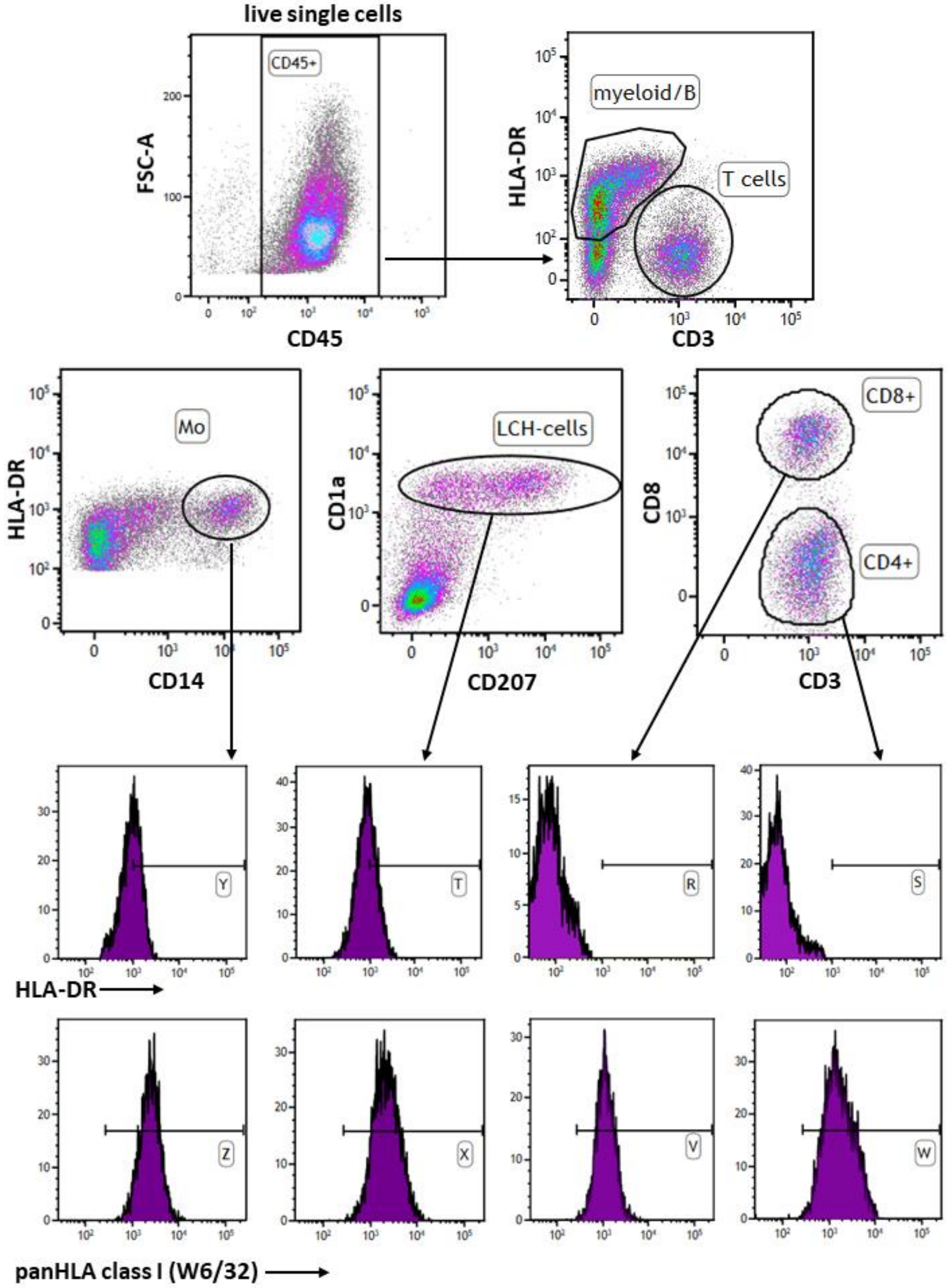

Figure S5. Frequency of HLA-DRB1*03 in LCH-patients from our cohort. SS, single-system LCH disease; MS, multisystem LCH disease; n, number of patients.

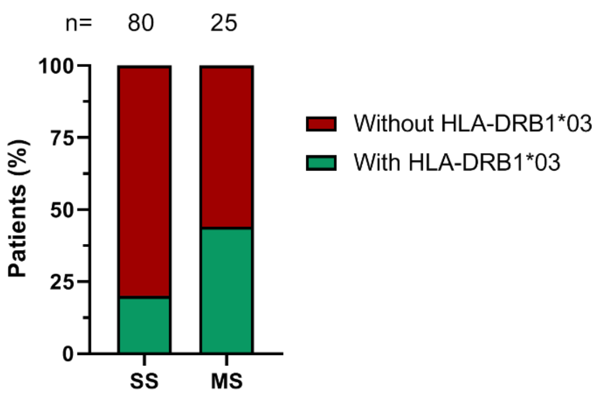

Figure S6. Distribution of LCH-lesional CD3⁺ T cell:CD1a⁺ LCH-cell ratios in first-disease onset tissue biopsies of n=101 LCH-patients.

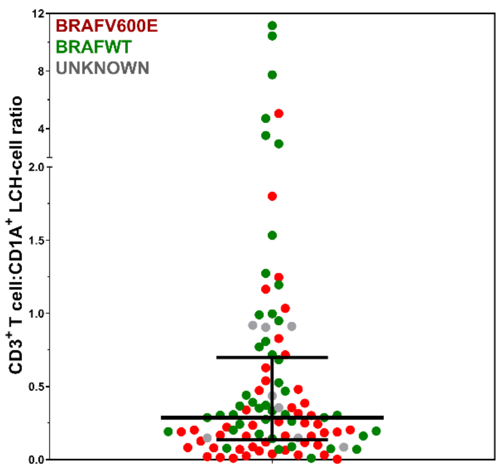

Figure S7. Distribution of CD3⁺ T cell:CD1a⁺ LCH-cell ratios in *BRAF*^{V600E} mutated (n=48) and *BRAF* wildtype (n=45) LCH-lesions.

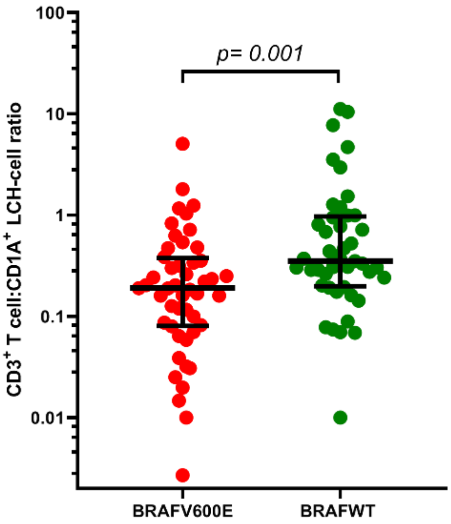

Figure S8. LCH-lesional CD3$^+$ and CD3$^+$CD8$^+$ T cell densities of *BRAF^{V600E}* mutated and *BRAF* wildtype LCH-patients according to the two additional analysis methods. (A) Percentage of *BRAF^{V600E}* mutated and *BRAF* wildtype patients of total patients with 1+ (n=25), 2+ (n=29), 3+ (n=15) or 4+ (n=6) CD8$^+$ T cell density score and total patients with 1+ (n=13), 2+ (n=29), 3+ (n=23) or 4+ (n=10) CD3$^+$ T cell density score. To maintain sufficient statistical power, patients with 1+ or 2+ ("LOW") T cell density scores were compared with patients with 3+ or 4+ ("HIGH") T cell density scores. (B) Distribution of calculated Purple area/Green area and Purple&Red area/Green area ratios for *BRAF^{V600E}* mutated (n=24) and *BRAF* wildtype (n=22) LCH-patients.

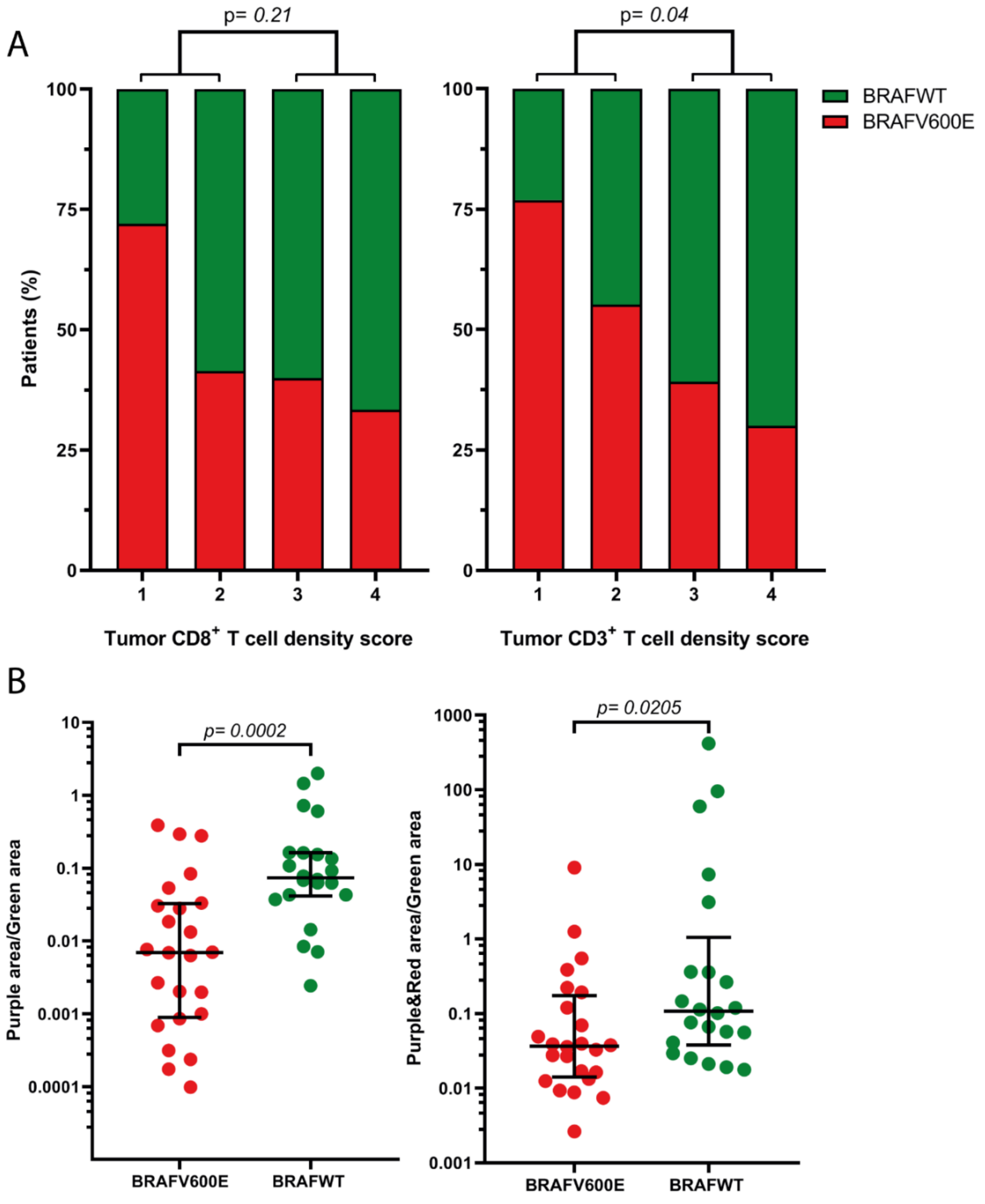

Figure S9. Event-free survival of *BRAF*^{V600E} mutated LCH-patients with and without HLA-A*03:01 and/or HLA-A*11:01 genotypes.

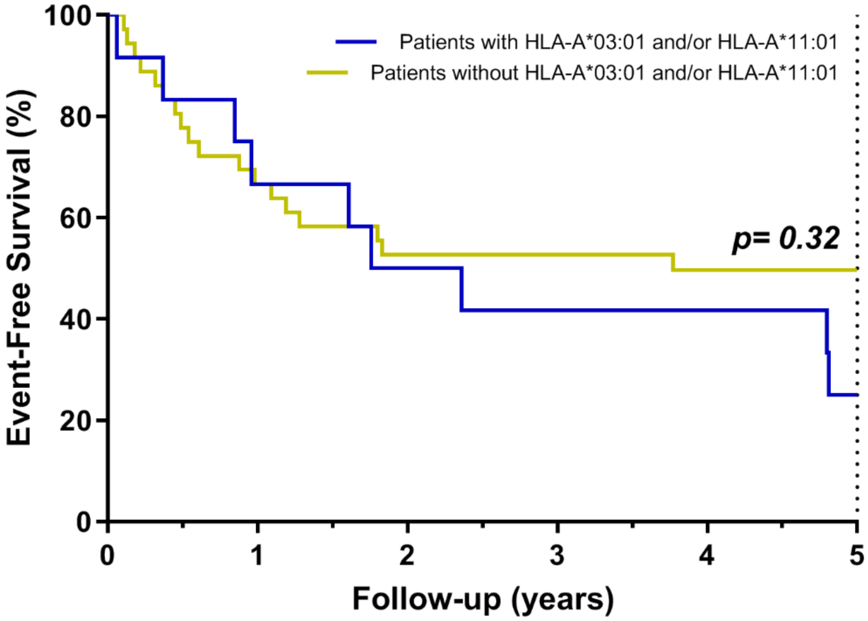

Figure S10. HLA subtype expression by untransduced and *BRAF*^{V600E} transduced SB and MLA EBV-LCL.

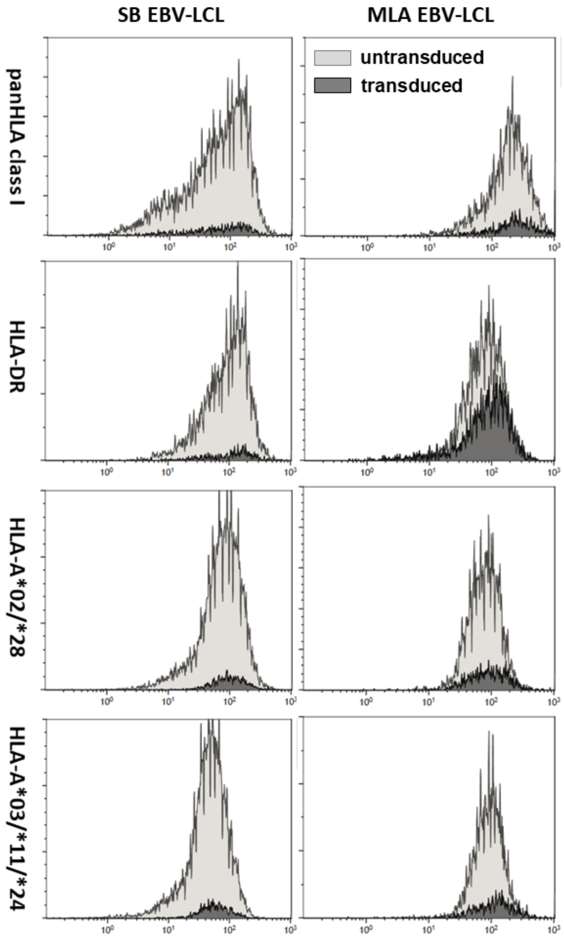

Figure S11. PD-1 expression by LCH-lesional CD3$^+$CD8$^-$ and CD3$^+$CD8$^+$ T cells. Top and middle images: CD3, CD8 and PD-1 expression by T cells present in a *BRAF*^{V600E} positive LCH-biopsy. Images were taken at 800x magnification. Bottom left image: CD8$^+$ T cells in purple, autofluorescent tissue in gray (signal recorded in the DAPI_Q channel, without the tissue being stained for DAPI). Image taken at 600x magnification. The tissue area shown in the top and middle images is framed. Bottom right: CD1a (green)/CD3 (not shown)/CD8 (purple) immunostained tissue section of the same *BRAF*^{V600E} positive LCH-biopsy. Although this image is captured from (a different area from) a different tissue section than the CD3/CD8/PD-1 immunostained tissue section, the CD1a$^+$ multinucleated giant cells (MGCs) and LCH-cells observed in the bottom right image can clearly be recognized in the gray autofluorescent tissue that is present in the bottom left image.

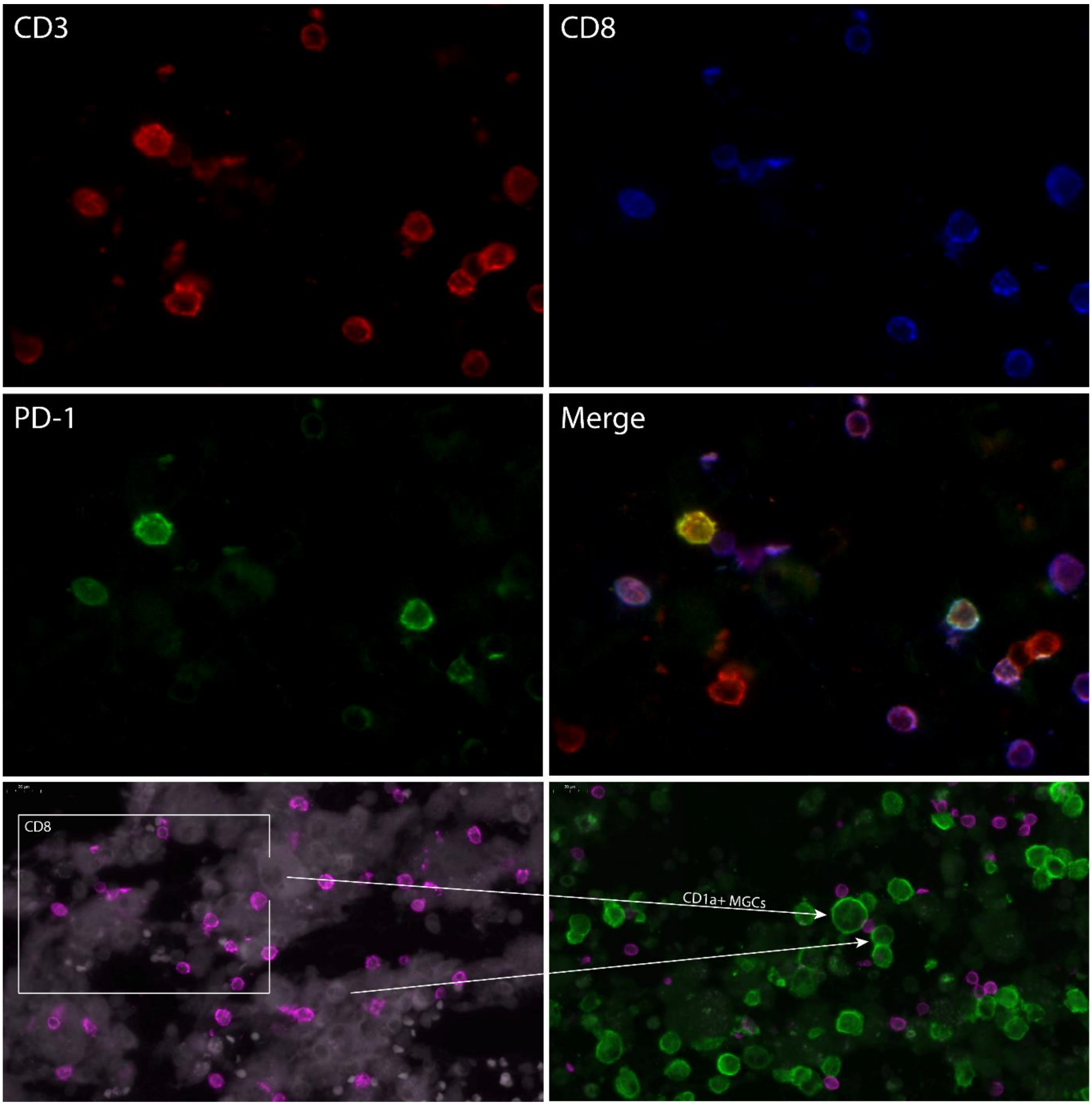

Figure S12. Rare Granzyme B and Perforin expression by LCH-lesional CD8$^+$ T cells. (A) CD1a/CD3/CD8 immunostained tissue section of a *BRAF^{V600E}* mutated LCH-biopsy. Image taken at 200x magnification. (B) CD8/Granzyme B/Perforin immunostained consecutively cut tissue section of the same *BRAF^{V600E}* mutated LCH-biopsy. Image taken at 200x magnification of the same tissue area (recognizable in the autofluorescent signal) as depicted in panel A. The tissue area shown in panel C-F is framed. (C-F) Rare Granzyme B (E) and Perforin (F) co-expression by CD8$^+$ (D) T cells, suggesting that the majority of the LCH-lesional CD8$^+$ T cells are dysfunctional.

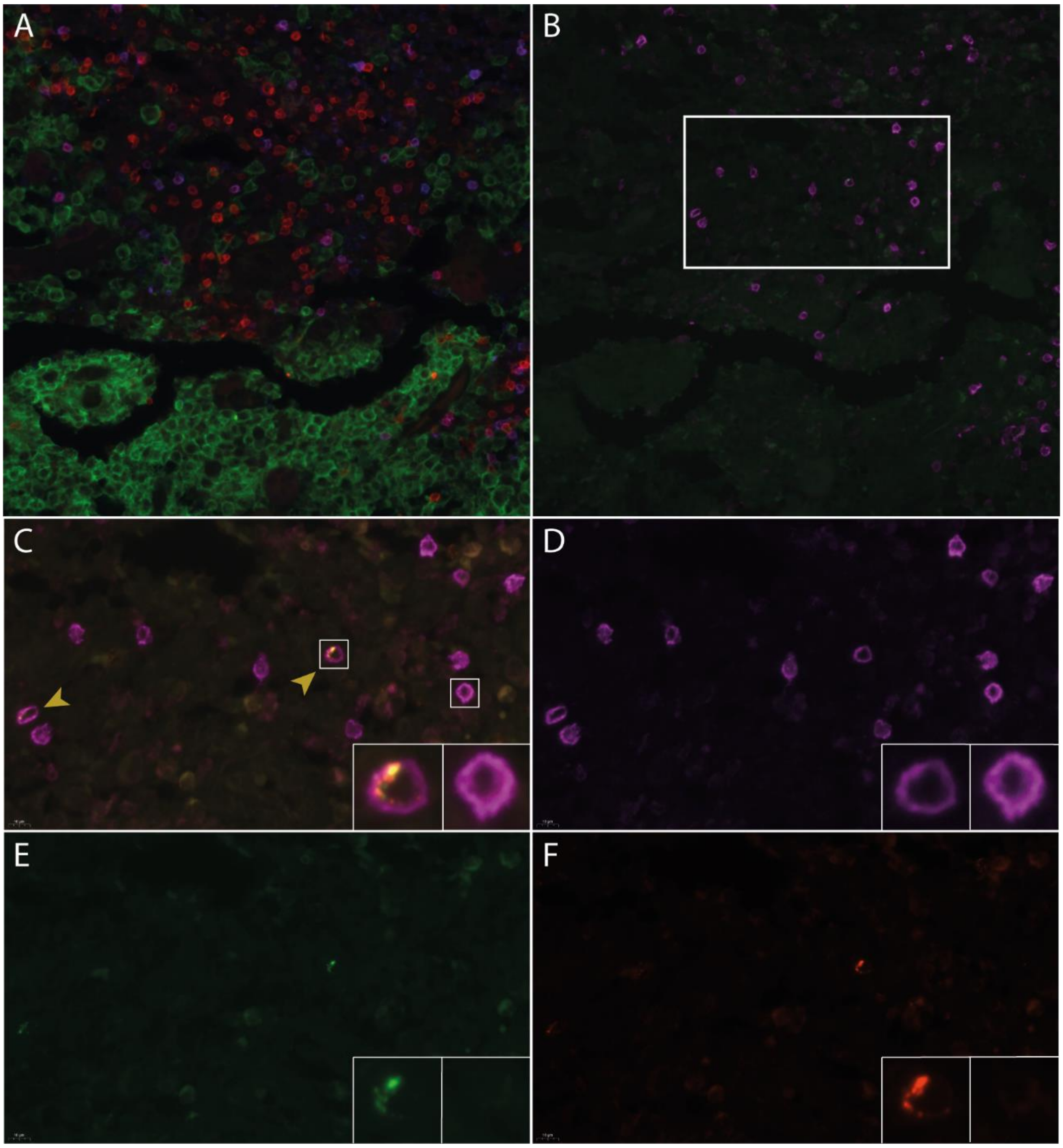

SUPPLEMENTARY TABLES

Table S1. PRM parameters used for the acquisition of eluted (light) and heavy labelled peptides. Peptide sequences are shown and heavy isotopes are depicted in bold. Masses are given as mass-to-charge ratio in m/z. The normalized collision energy is in %.

Peptide sequence	Peptide label	Precursor charge	Q1 mass	Fragment mass	Fragment charge	Fragment ion	Collision energy
KIGDFGLATE	Light	2	525,8	249,1	1	y2	20
				320,1	1	y3	20
				731,4	1	b7	20
				802,4	1	b8	20
				903,5	1	b9	20
				452,3	2	b9	20
**K**IGDFGLATE	Heavy	2	529,8	249,1	1	y2	20
				320,1	1	y3	20
				731,4	1	b7	20
				802,4	1	b8	20
				903,5	1	b9	20
				452,3	2	b9	20
KIGDFGLATV	Light	2	510,8	219,1	1	y2	23
				731,4	1	b7	23
				802,4	1	b8	23
				903,5	1	b9	23
				452,3	2	b9	23
KIGDFGL**A**TV	Heavy	2	512,8	219,1	1	y2	23
				731,4	1	b7	23
				806,5	1	b8	23
				907,5	1	b9	23
				454,3	2	b9	23
KIGDFGLATEK	Light	2	589,8	377,2	1	y3	32
				448,2	1	y4	32
				618,3	1	y6	32
				765,4	1	y7	32
				937,5	1	y9	32
				1051	1	y10	32
KIGDFG**L**ATEK	Heavy	2	593,3	377,2	1	y3	32
				448,2	1	y4	32
				625,4	1	y6	32
				772,4	1	y7	32
				944,5	1	y9	32
				1058	1	y10	32
KIGDFGLATEK	Light	3	393,6	377,2	1	y3	14
				448,2	1	y4	14
				224,6	2	y4	14
				561,3	1	y5	14
				618,3	1	c6-17	14
				731,4	1	b7	14
KIGDFG**L**ATEK	Heavy	3	395,9	377,2	1	y3	14
				448,2	1	y4	14
				224,6	2	y4	14
				568,3	1	y5	14
				618,3	1	c6-17	14
				738,4	1	b7	14
KIGDFGLATVK	Light	2	574,8	347,2	1	y3	32
				418,3	1	y4	32
				734,4	1	y7	32
				907,5	1	y9	32
				1021	1	y10	32
KIGDFGLAT**V**K	Heavy	2	577,8	353,2	1	y3	32
				424,3	1	y4	32
				741,5	1	y7	32
				913,5	1	y9	32
				1027	1	y10	32
KIGDFGLATVK	Light	3	383,6	418,3	1	y4	14
				209,6	2	y4	14
				531,4	1	y5	14
				266,2	2	y5	14
				618,3	1	b6	14
				731,4	1	b7	14
KIGDFGLAT**V**K	Heavy	3	385,6	424,3	1	y4	14
				212,6	2	y4	14
				537,4	1	y5	14
				269,2	2	y5	14
				618,3	1	b6	14
				731,4	1	b7	14

Table S2. Results of the HLA association analyses, comparing HLA genotype data from n=94 Dutch LCH-patients to the HLA genotypes of n=5604 Dutch blood donors reflecting the HLA genotype of the Dutch population.

	Cases			Controls			Woolf-Haldane	95% C.I.		Fisher's Exact (2-tailed)		
Antigen	pos	neg	perc.	pos	neg	perc.	Odds Ratio	lower	upper	P	P (Sidak)	P (stand.)
A*01	30	64	31,9%	1887	3717	33,7%	0,93	0,603	1,438	0,8259	1,0000	0,5000
A*02	54	40	57,4%	2745	2859	49,0%	1,40	0,930	2,112	0,1185	0,9197	0,5000
A*03	25	69	26,6%	1640	3964	29,3%	0,89	0,561	1,401	0,6478	1,0000	0,5000
A*11	8	86	8,5%	649	4955	11,6%	0,75	0,369	1,524	0,4184	1,0000	0,5000
A*23	3	91	3,2%	134	5470	2,4%	1,56	0,528	4,587	0,4943	1,0000	0,5000
A*24	20	74	21,3%	921	4683	16,4%	1,40	0,853	2,292	0,2081	0,9906	0,5000
A*25	1	93	1,1%	122	5482	2,2%	0,72	0,142	3,639	0,7242	1,0000	0,5000
A*26	2	92	2,1%	248	5356	4,4%	0,58	0,165	2,059	0,4416	1,0000	0,5000
A*29	3	91	3,2%	299	5305	5,3%	0,68	0,232	1,983	0,4874	1,0000	0,5000
A*30	2	92	2,1%	276	5328	4,9%	0,52	0,147	1,840	0,3288	0,9997	0,5000
A*31	9	85	9,6%	314	5290	5,6%	1,87	0,947	3,689	0,1105	0,9039	0,5000
A*32	6	88	6,4%	355	5249	6,3%	1,08	0,486	2,423	1,0000	1,0000	0,5000
A*33	2	92	2,1%	113	5491	2,0%	1,31	0,367	4,656	0,7149	1,0000	0,5000
A*34	0	94	0,0%	17	5587	0,3%	1,69	0,101	28,300	1,0000	1,0000	0,5000
A*36	0	94	0,0%	9	5595	0,2%	3,12	0,180	53,936	1,0000	1,0000	0,5000
A*66	2	92	2,1%	33	5571	0,6%	4,49	1,223	16,516	0,1129	0,9090	0,5000
A*68	7	87	7,4%	517	5087	9,2%	0,84	0,398	1,786	0,7183	1,0000	0,5000
A*69	0	94	0,0%	9	5595	0,2%	3,12	0,180	53,936	1,0000	1,0000	0,5000
A*74	0	94	0,0%	5	5599	0,1%	5,39	0,296	98,119	1,0000	1,0000	0,5000
A*80	0	94	0,0%	2	5602	0,0%	11,86	0,565	248,675	1,0000	1,0000	0,5000
B*07	22	68	24,4%	1391	4213	24,8%	0,99	0,615	1,608	1,0000	1,0000	0,5000
B*08	25	65	27,8%	1436	4168	25,6%	1,13	0,712	1,792	0,6278	1,0000	0,5000
B*13	2	88	2,2%	229	5375	4,1%	0,66	0,187	2,342	0,5870	1,0000	0,5000
B14	4	86	4,4%	217	5387	3,9%	1,29	0,495	3,356	0,7792	1,0000	0,5000
B62	15	75	16,7%	837	4732	15,0%	1,16	0,668	2,014	0,6557	1,0000	0,5000
B63	0	90	0,0%	44	5525	0,8%	0,69	0,042	11,226	1,0000	1,0000	0,5000
B75	0	90	0,0%	16	5553	0,3%	1,86	0,111	31,235	1,0000	1,0000	0,5000
B76	0	90	0,0%	1	5568	0,0%	20,51	0,830	506,902	1,0000	1,0000	0,5000
B77	0	90	0,0%	9	5560	0,2%	3,23	0,187	55,986	1,0000	1,0000	0,5000
B*18	8	82	8,9%	414	5190	7,4%	1,29	0,632	2,632	0,5422	1,0000	0,5000
B*27	8	82	8,9%	405	5199	7,2%	1,32	0,647	2,696	0,5358	1,0000	0,5000
B*35	8	82	8,9%	1090	4514	19,5%	0,43	0,210	0,867	0,0100	0,3301	0,5000
B*37	2	88	2,2%	181	5423	3,2%	0,84	0,238	2,993	1,0000	1,0000	0,5000
B*38	2	88	2,2%	186	5418	3,3%	0,82	0,232	2,909	0,7703	1,0000	0,5000
B*39	4	86	4,4%	229	5375	4,1%	1,22	0,468	3,172	0,7864	1,0000	0,5000
B60	14	76	15,6%	676	4832	12,3%	1,35	0,768	2,387	0,3325	1,0000	0,5000
B61	3	87	3,3%	198	5310	3,6%	1,07	0,364	3,144	1,0000	1,0000	0,5000
B*41	6	84	6,7%	86	5518	1,5%	4,91	2,149	11,205	0,0032	0,1191	0,4736
B*42	0	90	0,0%	9	5595	0,2%	3,25	0,188	56,338	1,0000	1,0000	0,5000
B*44	18	72	20,0%	1257	4347	22,4%	0,88	0,527	1,476	0,7021	1,0000	0,5000
B*45	1	89	1,1%	60	5544	1,1%	1,54	0,300	7,866	0,6236	1,0000	0,5000
B*46	0	90	0,0%	9	5595	0,2%	3,25	0,188	56,338	1,0000	1,0000	0,5000
B*47	1	89	1,1%	29	5575	0,5%	3,17	0,606	16,556	0,3807	1,0000	0,5000
B*48	1	89	1,1%	5	5599	0,1%	17,06	2,772	105,043	0,0912	0,9782	0,5000
B*49	0	90	0,0%	84	5520	1,5%	0,36	0,022	5,864	0,6444	1,0000	0,5000
B50	4	86	4,4%	84	5520	1,5%	3,40	1,286	8,981	0,0499	0,8709	0,5000
B*51	13	77	14,4%	590	5014	10,5%	1,48	0,825	2,653	0,2263	1,0000	0,5000
B*52	2	88	2,2%	50	5554	0,9%	3,11	0,858	11,255	0,1983	0,9999	0,5000
B*53	1	89	1,1%	40	5564	0,7%	2,30	0,445	11,907	0,4808	1,0000	0,5000
B*54	0	90	0,0%	2	5602	0,0%	12,38	0,590	259,743	1,0000	1,0000	0,5000
B*55	0	90	0,0%	239	5365	4,3%	0,12	0,008	2,000	0,0331	0,7401	0,5000
B*56	0	90	0,0%	77	5527	1,4%	0,39	0,024	6,406	0,6354	1,0000	0,5000
B*57	5	85	5,6%	368	5236	6,6%	0,91	0,383	2,179	1,0000	1,0000	0,5000
B*58	2	88	2,2%	93	5511	1,7%	1,67	0,466	5,950	0,6628	1,0000	0,5000
B*67	0	90	0,0%	1	5603	0,0%	20,64	0,835	510,088	1,0000	1,0000	0,5000
B*71	0	90	0,0%	19	5550	0,3%	1,57	0,094	26,248	1,0000	1,0000	0,5000
B72	1	89	1,1%	26	5543	0,5%	3,51	0,668	18,406	0,3520	1,0000	0,5000
B*73	0	90	0,0%	2	5602	0,0%	12,38	0,590	259,743	1,0000	1,0000	0,5000
B*78	0	90	0,0%	3	5601	0,1%	8,84	0,453	172,438	1,0000	1,0000	0,5000
B*81	0	90	0,0%	3	5601	0,1%	8,84	0,453	172,438	1,0000	1,0000	0,5000
C*01	4	89	4,3%	323	5281	5,8%	0,82	0,316	2,130	0,8204	1,0000	0,5000
C*02	9	84	9,7%	575	5029	10,3%	0,98	0,500	1,932	1,0000	1,0000	0,5000

C*03	28	65	30,1%	1665	3939	29,7%	1,03	0,661	1,604	0,9094	1,0000	0,5000
C*04	12	81	12,9%	1287	4317	23,0%	0,51	0,283	0,936	0,0240	0,2888	0,5000
C*05	12	81	12,9%	764	4840	13,6%	0,97	0,533	1,770	1,0000	1,0000	0,5000
C*06	12	81	12,9%	898	4706	16,0%	0,80	0,441	1,463	0,4775	0,9999	0,5000
C*07	52	41	55,9%	3140	2464	56,0%	0,99	0,659	1,497	1,0000	1,0000	0,5000
C*08	5	88	5,4%	233	5371	4,2%	1,43	0,598	3,417	0,5948	1,0000	0,5000
C*12	10	83	10,8%	466	5138	8,3%	1,39	0,724	2,650	0,3487	0,9975	0,5000
C*14	5	88	5,4%	117	5487	2,1%	2,90	1,203	7,001	0,0485	0,5014	0,5000
C*15	7	86	7,5%	287	5317	5,1%	1,60	0,753	3,414	0,3366	0,9968	0,5000
C*16	6	87	6,5%	343	5261	6,1%	1,14	0,509	2,543	0,8268	1,0000	0,5000
C*17	6	87	6,5%	97	5507	1,7%	4,20	1,845	9,543	0,0065	0,0868	0,3396
C*18	1	92	1,1%	2	5602	0,0%	36,34	4,751	277,998	0,0482	0,4991	0,5000
DRB1*01	12	69	14,8%	1234	4370	22,0%	0,64	0,348	1,167	0,1368	0,8725	0,5000
DR17	24	57	29,6%	1524	3990	27,6%	1,12	0,693	1,796	0,7078	1,0000	0,5000
DR18	0	81	0,0%	8	5506	0,1%	3,97	0,227	69,440	1,0000	1,0000	0,5000
DRB1*04	30	51	37,0%	1557	4047	27,8%	1,54	0,980	2,418	0,0798	0,6878	0,5000
DRB1*07	16	65	19,8%	1098	4506	19,6%	1,03	0,600	1,780	1,0000	1,0000	0,5000
DRB1*08	5	76	6,2%	361	5243	6,4%	1,04	0,436	2,494	1,0000	1,0000	0,5000
DRB1*09	4	77	4,9%	160	5444	2,9%	1,97	0,752	5,161	0,2969	0,9928	0,5000
DRB1*10	2	79	2,5%	132	5472	2,4%	1,30	0,364	4,628	0,7166	1,0000	0,5000
DRB1*11	13	68	16,0%	930	4674	16,6%	0,99	0,550	1,782	1,0000	1,0000	0,5000
DRB1*12	2	79	2,5%	210	5394	3,7%	0,81	0,227	2,859	0,7701	1,0000	0,5000
DRB1*13	19	62	23,5%	1393	4211	24,9%	0,94	0,565	1,573	0,8970	1,0000	0,5000
DRB1*14	5	76	6,2%	373	5231	6,7%	1,01	0,421	2,408	1,0000	1,0000	0,5000
DRB1*15	15	66	18,5%	1356	4248	24,2%	0,73	0,419	1,273	0,2948	0,9925	0,5000
DRB1*16	6	75	7,4%	165	5439	2,9%	2,83	1,251	6,399	0,0344	0,3871	0,5000
DQB1*02	35	46	43,2%	2204	3400	39,3%	1,18	0,758	1,829	0,4933	0,9914	0,5000
DQ7	24	57	29,6%	1641	3894	29,6%	1,01	0,628	1,628	1,0000	1,0000	0,5000
DQ8	19	62	23,5%	1073	4462	19,4%	1,30	0,777	2,166	0,3951	0,9704	0,5000
DQ9	8	73	9,9%	504	5031	9,1%	1,15	0,564	2,360	0,8448	1,0000	0,5000
DQB1*04	4	77	4,9%	340	5264	6,1%	0,90	0,345	2,337	1,0000	1,0000	0,5000
DQB1*05	27	54	33,3%	1840	3764	32,8%	1,03	0,650	1,638	0,9057	1,0000	0,5000
DQB1*06	31	50	38,3%	2470	3134	44,1%	0,79	0,506	1,239	0,3123	0,9272	0,5000

missing:	
HLA-A	0
HLA-B	4
HLA-C	1
HLA-DR	13
HLA-DQ	13

Table S3. Hardy-Weinberg Equilibrium testing of HLA genotype data from n=94 Dutch LCH-patients.

	homozygotes	heterozygotes	overall
HLA-A	0.4881	0.7460	n.c.
HLA-B	0.8287	0.9483	n.c.
HLA-C	0.6619	0.8462	n.c.
HLA-DR	0.8711	0.9528	n.c.
HLA-DQ	0.8593	0.9336	0.0730

n.c. = not calculated (too many parameters)

Table S4. Frequency of HLA subtypes that were described to be associated with specific LCH clinical presentations in patients from our cohort. [a], control phenotype frequencies calculated from published gene frequencies for 5604 healthy Dutch controls.

HLA antigen	Selected LC patient group	Selected Caucasian patients	Frequency in Dutch controls (%)[a]
**Cw7**	Total Dutch patients, 52/93 (56%), P = 1.000 (NS), OR = 0.993 (0.659-1.497)	Total Dutch Caucasians, 49/84 (58%), P = 0.740 (NS), OR = 1.094 (0.709-1.689)	56%
**Cw7**	Dutch Caucasians with single bone disease or skin disease, 27/50 (54%), P = 0.777 (NS), OR = 0.918 (0.528-1.597)	Dutch Caucasians with single bone disease, 20/38 (53%), P = 0.744 (NS), OR = 0.870 (0.463-1.634)	56%
**B7 or B8**	Total Dutch Caucasians, 43/85 (51%), P = 0.226 (NS), OR = 1.322 (0.864-2.025)	Dutch Caucasians with single bone lesions, 18/38 (47%), P = 0.743 (NS), OR = 1.166 (0.620-2.191)	44%
**DR4 and/or Cw7**	Total Dutch Caucasians, 54/72 (75%), P = 0.600 (NS), OR = 1.171 (0.689-1.991)	Dutch Caucasians with single bone lesions, 28/37 (76%), P = 0.715 (NS), OR = 1.193 (0.571-2.491)	72%
**DR4**	Total Dutch Caucasians: 27/72 (38%), P = 0.085 (NS), OR = 1.571 (0.975-2.531)	Dutch Caucasians with single bone lesions, 15/37 (41%), P = 0.097 (NS), OR = 1.790 (0.935-3.428)	28%

Additional data

HLA antigen	Selected LC patient group	Selected LC patient group	Frequency in Dutch controls (%)[a]
**Cw7**	Dutch patients with single bone disease or skin disease, 29/54 (54%), P = 0.784 (NS), OR = 0.908 (0.533-1.546)	Dutch patients with single bone disease, 22/41 (54%), P = 0.755 (NS), OR = 0.905 (0.493-1.664)	56%
**B7 or B8**	Total Dutch patients, 44/94 (47%), P = 0.600 (NS), OR = 1.138 (0.758-1.709)	Dutch patients with single bone lesions, 19/41 (46%), P = 0.754 (NS), OR = 1.120 (0.609-2.058)	44%
**B7 and/or B8**	Total Dutch Caucasians, 44/85 (52%), P = 0.444 (NS), OR = 1.207 (0.788-1.849)	Dutch Caucasians with single bone lesions, 19/38 (50%), P = 0.746 (NS), OR = 1.126 (0.600-2.114)	47%
**B7 and/or B8**	Total Dutch patients, 46/94 (49%), P = 0.755 (NS), OR = 1.079 (0.720-1.620)	Dutch patients with single bone lesions, 20/41 (49%), P = 0.876 (NS), OR = 1.074 (0.585-1.970)	47%
**DR4 and/or Cw7**	Total Dutch patients, 59/81 (73%), P = 0.901 (NS), OR = 1.051 (0.645-1.714)	Dutch patients with single bone lesions, 31/40 (78%), P = 0.484 (NS), OR = 1.318 (0.637-2.730)	72%
**DR4**	Total Dutch patients, 30/81 (37%), P = 0.080 (NS), OR = 1.539 (0.980-2.418)	Total Dutch patients with single bone lesions: 17/40 (43%), P = 0.050 (NS), OR = 1.935 (1.039-3.603)	28%

Table S5. Predicted HLA binding affinities of *BRAF*^{V600E} and *BRAF* wildtype protein-derived peptides according to NetMHC 4.0 software. Peptides highlighted in green are generated by the human proteasome according to NetCHOP 3.1 software. Peptides with a predicted IC_{50} of >500nM are considered non-binders.

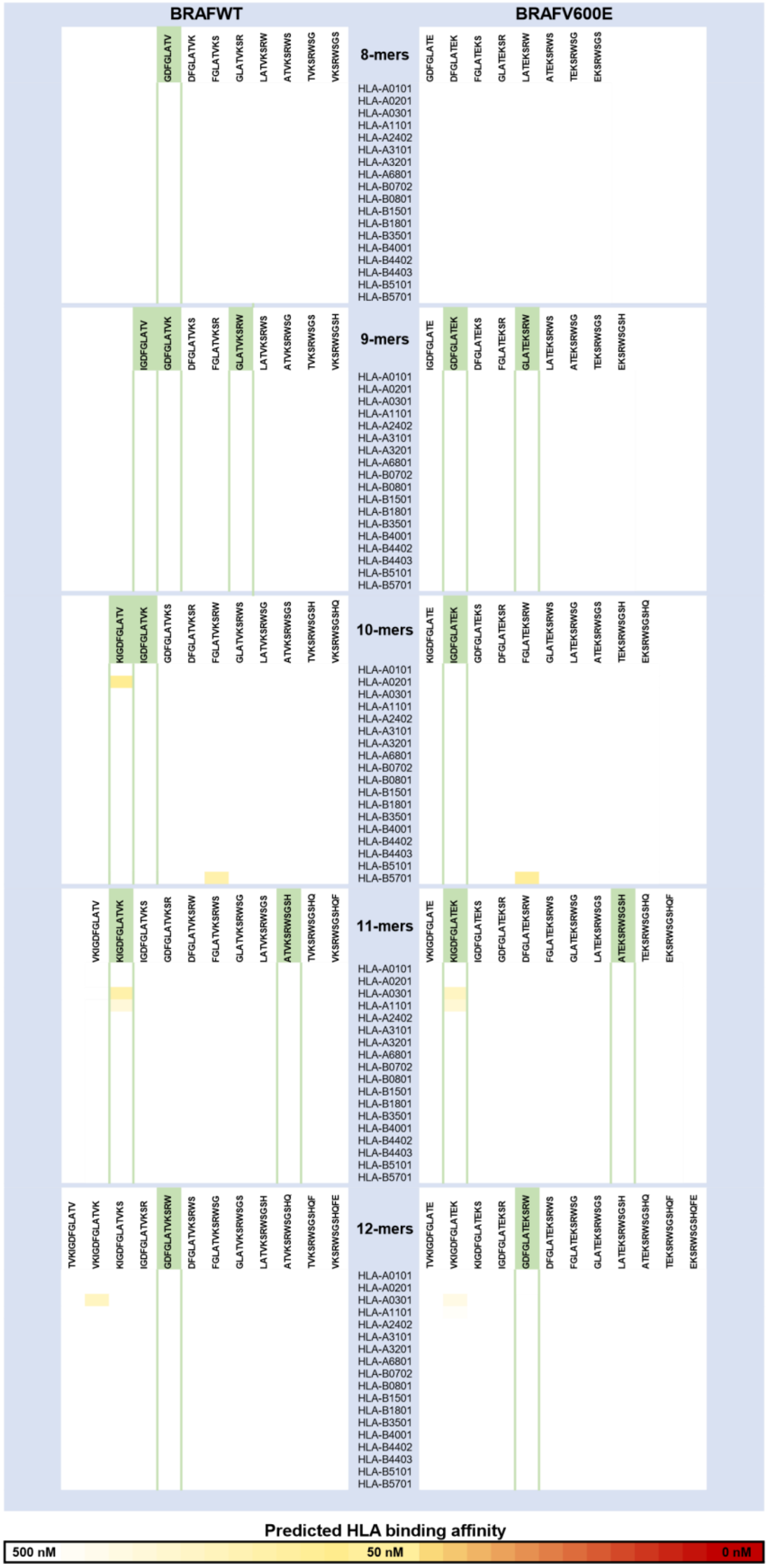

Table S6. Characteristics of *BRAF*^{V600E} mutated LCH-patients with known high-resolution HLA genotype. SS, single-system LCH disease; MS RO-, multisystem LCH disease without risk organ (bone marrow, liver and/or spleen) involvement; MS RO+, multisystem LCH disease with risk organ involvement; FDO, first disease onset.

	All patients	Patients with HLA-A*03:01 and/or HLA-A*11:01	Patients without HLA-A*03:01 and/or HLA-A*11:01	P-value
**Patients**	48	12 (25%)	36 (75%)	
**Gender**				
**Male**	26 (54%)	4 (33%)	22 (61%)	0.11
**Female**	22 (46%)	8 (67%)	14 (39%)	
**Age distribution**				
**Pediatric patients**	42 (87.5%)	11 (92%)	31 (86%)	1
**Adult patients**	6 (12.5%)	1 (8%)	5 (14%)	
**Disease extension**				
**SS**	33 (69%)	8 (67%)	25 (69%)	1
**MS RO-**	6 (12.5%)	2 (17%)	4 (11%)	0.63
**MS RO+**	9 (18.5%)	2 (17%)	7 (19%)	1
**Chemotherapy for FDO**	21 (44%)	6 (50%)	15 (42%)	0.74
**Follow-up (median)**	9.5 years	7.7 years	9.6 years	0.80

Table S7. Extended HLA genotypes of the cell lines that were used in the targeted peptidomics experiments. JY, JY EBV-LCL; MLA, MLA EBV-LCL; SB, SB EBV-LCL; HT29, colon carcinoma cell line harboring the heterozygous *BRAF*^{V600E} mutation.

Cell line	A-locus	B-locus	C-locus	DRB-locus	DQB-locus	DPB-locus
**JY**	A*02:01, A*02:01	B*07:02, B*07:02	C*07:02, C*07:02	DR4, DR13	DQB1, DQB3	DPB1*02:01, DPB1*02:01
**MLA**	A*02:01, A*11:01	B*15:01, B*27:05	C*02:02, C*03:04	DRB1*07, DRB1*14:54/14:01/14:07+	DQB1*02, DQB1*06:02	DPB1*02:01, DPB1*04:01
**SB**	A*02:01, A*03:01	B*15:01, B*27:05	C*01, C*03:04	DRB1*04:01, DRB1*13:01	DQB1*03:02, DQB1*06:03	DPB1*02:01:02, DPB1*10:01
**HT29**	A*01, A*24	B*35, B*44:03:01	C*04, C*04	DRB1*04:02, DRB1*07:01	DQB1*02:01, DQB1*03:02	DPB1*04:01, DPB1*04:01

Table S8. Peptides detected using data-dependent acquisition-based peptidomics in the HLA class I peptide pools isolated from the mock transduced JY and MLA EBV-LCL. High and medium confident (percolator) 8-12mer peptides are shown. A mascot ion score of >35 was set.

Table S8 is included as a separate Excel file.
